# *In vitro* generated antibodies guide thermostable ADDomer nanoparticle design for nasal vaccination and passive immunization against SARS-CoV-2

**DOI:** 10.1093/abt/tbad024

**Published:** 2023-10-17

**Authors:** Dora Buzas, Adrian H Bunzel, Oskar Staufer, Emily J Milodowski, Grace L Edmunds, Joshua C Bufton, Beatriz V Vidana Mateo, Sathish K N Yadav, Kapil Gupta, Charlotte Fletcher, Maia K Williamson, Alexandra Harrison, Ufuk Borucu, Julien Capin, Ore Francis, Georgia Balchin, Sophie Hall, Mirella V Vega, Fabien Durbesson, Srikanth Lingappa, Renaud Vincentelli, Joe Roe, Linda Wooldridge, Rachel Burt, Ross J L Anderson, Adrian J Mulholland, Bristol UNCOVER Group, Jonathan Hare, Mick Bailey, Andrew D Davidson, Adam Finn, David Morgan, Jamie Mann, Joachim Spatz, Frederic Garzoni, Christiane Schaffitzel, Imre Berger

**Affiliations:** Max Planck Bristol Centre for Minimal Biology, University of Bristol, Bristol BS8 1TS, UK; School of Biochemistry, University of Bristol, Bristol BS8 1TD, UK; School of Biochemistry, University of Bristol, Bristol BS8 1TD, UK; Max Planck Bristol Centre for Minimal Biology, University of Bristol, Bristol BS8 1TS, UK; Leibniz Institute for New Materials, Helmholtz Institute for Pharmaceutical Research and Center for Biophysics, Saarland University, Saarbrücken 66123, Germany; Bristol Veterinary School, University of Bristol, Bristol BS40 5DU UK; Bristol Veterinary School, University of Bristol, Bristol BS40 5DU UK; School of Biochemistry, University of Bristol, Bristol BS8 1TD, UK; Bristol Veterinary School, University of Bristol, Bristol BS40 5DU UK; School of Biochemistry, University of Bristol, Bristol BS8 1TD, UK; School of Biochemistry, University of Bristol, Bristol BS8 1TD, UK; Imophoron Ltd, Science Creates Old Market, Midland Rd, Bristol BS2 0JZ UK; School of Biochemistry, University of Bristol, Bristol BS8 1TD, UK; School of Cellular and Molecular Medicine, University of Bristol, Bristol, BS8 1TD, UK; School of Biochemistry, University of Bristol, Bristol BS8 1TD, UK; School of Biochemistry, University of Bristol, Bristol BS8 1TD, UK; School of Biochemistry, University of Bristol, Bristol BS8 1TD, UK; Bristol Veterinary School, University of Bristol, Bristol BS40 5DU UK; School of Biochemistry, University of Bristol, Bristol BS8 1TD, UK; School of Biochemistry, University of Bristol, Bristol BS8 1TD, UK; School of Biochemistry, University of Bristol, Bristol BS8 1TD, UK; Architecture et Fonction des Macromolécules Biologiques, UMR 7257, CNRS, Aix-Marseille Université, Marseille, France; School of Biochemistry, University of Bristol, Bristol BS8 1TD, UK; Architecture et Fonction des Macromolécules Biologiques, UMR 7257, CNRS, Aix-Marseille Université, Marseille, France; Bristol Veterinary School, University of Bristol, Bristol BS40 5DU UK; Bristol Veterinary School, University of Bristol, Bristol BS40 5DU UK; Bristol Veterinary School, University of Bristol, Bristol BS40 5DU UK; School of Biochemistry, University of Bristol, Bristol BS8 1TD, UK; School of Chemistry, University of Bristol, Bristol BS8 1TS, UK; Bristol University COVID-19 Emergency Research Group, Bristol BS8 1TH, UK; School of Cellular and Molecular Medicine, University of Bristol, Bristol, BS8 1TD, UK; Bristol Veterinary School, University of Bristol, Bristol BS40 5DU UK; Imophoron Ltd, Science Creates Old Market, Midland Rd, Bristol BS2 0JZ UK; Bristol University COVID-19 Emergency Research Group, Bristol BS8 1TH, UK; Children's Vaccine Centre, Bristol Medical School, Bristol BS2 8EF UK; Imophoron Ltd, Science Creates Old Market, Midland Rd, Bristol BS2 0JZ UK; Bristol Veterinary School, University of Bristol, Bristol BS40 5DU UK; Max Planck Bristol Centre for Minimal Biology, University of Bristol, Bristol BS8 1TS, UK; Max Planck Institute for Medical Research, Heidelberg 69120, Germany; School of Cellular and Molecular Medicine, University of Bristol, Bristol, BS8 1TD, UK; School of Biochemistry, University of Bristol, Bristol BS8 1TD, UK; Bristol University COVID-19 Emergency Research Group, Bristol BS8 1TH, UK; Max Planck Bristol Centre for Minimal Biology, University of Bristol, Bristol BS8 1TS, UK; School of Biochemistry, University of Bristol, Bristol BS8 1TD, UK; School of Chemistry, University of Bristol, Bristol BS8 1TS, UK; Bristol University COVID-19 Emergency Research Group, Bristol BS8 1TH, UK

**Keywords:** pandemic preparedness, ADDomer, protein nanoparticle design, in vitro selection (Ribosome Display), Gigabody superbinder, synthetic vaccine, electron cryo-microscopy (cryo-EM), respiratory viral diseases

## Abstract

**Background:**

Due to COVID-19, pandemic preparedness emerges as a key imperative, necessitating new approaches to accelerate development of reagents against infectious pathogens.

**Methods:**

Here, we developed an integrated approach combining synthetic, computational and structural methods with *in vitro* antibody selection and *in vivo* immunization to design, produce and validate nature-inspired nanoparticle-based reagents against severe acute respiratory syndrome coronavirus 2 (SARS-CoV-2).

**Results:**

Our approach resulted in two innovations: (i) a thermostable nasal vaccine called ADDoCoV, displaying multiple copies of a SARS-CoV-2 receptor binding motif derived epitope and (ii) a multivalent nanoparticle superbinder, called Gigabody, against SARS-CoV-2 including immune-evasive variants of concern (VOCs). *In vitro* generated neutralizing nanobodies and electron cryo-microscopy established authenticity and accessibility of epitopes displayed by ADDoCoV. Gigabody comprising multimerized nanobodies prevented SARS-CoV-2 virion attachment with picomolar EC_50_. Vaccinating mice resulted in antibodies cross-reacting with VOCs including Delta and Omicron.

**Conclusion:**

Our study elucidates Adenovirus-derived dodecamer (ADDomer)-based nanoparticles for use in active and passive immunization and provides a blueprint for crafting reagents to combat respiratory viral infections.

## Teaser

Statement of Significance: COVID-19 reminds us of the critical importance of pandemic preparedness, requiring sophisticated approaches to facilitate and accelerate reagent development against emerging threats. We described an integrated pipeline approach to rapidly craft in parallel ADDomer-based nanoparticles for vaccination and for passive immunization, applicable to many infectious diseases.

## INTRODUCTION

As of early 2023, the COVID-19 pandemic, caused by severe acute respiratory syndrome coronavirus 2 (SARS-CoV-2), continues to spread globally, with over 660 million confirmed cases and close to 7 million deaths reported worldwide according to the World Health Organization (WHO). The economic damage caused by the pandemic has been significant, with many countries having experienced severe economic downturns as a result of lockdowns and other non-pharmaceutical intervention measures implemented to slow the spread of the virus.

In an unprecedented effort, numerous COVID-19 vaccine candidates were developed at record speed [1–4], and several were authorized for emergency use or received full approval by regulatory agencies around the world [1]. Among these, prominent examples include Cominatry (Pfizer-BioNTech) and Spikevax (Moderna) mRNA vaccines [5–7], Vaxzevria (Oxford-AstraZeneca) and Jcovden (Johnson & Johnson) adenovirus vectored vaccines [8–11], and the Nuvaxovid (Novavax) protein subunit vaccine [12]. These vaccines have been shown to be effective in preventing severe COVID-19, with the mRNA vaccines exhibiting highest efficacy rates (~95%) although this is relatively short-lived [1].

In addition, monoclonal antibody treatments were rapidly rolled out for intravenous administration, including REGN-COV2 (Regeneron), bamlavinimab and etesevimab (Eli Lilly) AZDZ7442 (Astra Zeneca) and sotrovimab (GlaxoSmithKline and Vir Biotechnology), typically comprising one or two monoclonal antibody species neutralizing SARS-CoV-2 [13–15]. These received approval for the treatment of mild to moderate COVID-19 in adults and pediatric patients, with mixed results due to reduced effectiveness against rapidly evolving SARS-CoV-2 variants—bamlanivimab and etesevimab were withdrawn, while sotrovimab is no longer widely used.

All currently licensed COVID-19 vaccines require refrigeration to maintain their stability and potency and depend on a functioning cold-chain [16–18]. The same applies broadly to monoclonal antibody treatments [19]. This renders distribution and storage logistics challenging, especially in areas with limited access to refrigeration, which includes most developing nations with often remote or impoverished regions. Therefore, developing thermostable reagents not reliant on cold-chain storage and distribution, would greatly simplify the roll-out process and reduce costs, and thus increase accessibility globally.

Nanoparticle-based vaccines hold great promise for overcoming the limitations of current vaccine technologies [20–22]. Shortly before the pandemic, we introduced ADDomer (Adenovirus-derived dodecamer), a synthetic self-assembling protein nanoparticle platform for highly efficient vaccination by genetically encoded multiepitope display [23]. ADDomer derives from a single protein component of adenovirus, which forms pentons at the vertices of the viral capsid, providing a base for the attachment of the adenoviral fiber [24]. When produced recombinantly in isolation, 60 copies of this penton-base protomer spontaneously self-assemble in the test-tube into a dodecahedron comprising 12 pentons. ADDomer is stable at temperatures exceeding 50°C and can be stored at ambient temperature for prolonged periods, indicating that ADDomer-based vaccines and therapeutics could be potentially produced, stored and transported without the need for refrigeration [23]. Exposed loops on the ADDomer surface function as insertion sites for rationally selected immunogenic peptide epitopes [23, 25].

In this study, we set out to develop a thermostable ADDomer-based COVID-19 vaccine, with the potential to overcome limitations associated with the cold-chain challenge, while maintaining the advantages of ADDomer notably the ease of production of a single-component particle carrying genetically encoded antigens. We called this vaccine ADDoCoV (for ADDomer-COVID-19 vaccine). We validate our ADDoCoV design by near-atomic resolution electron cryo-microscopy (cryo-EM) and molecular dynamics (MD) in a hybrid approach. We confirm authenticity and accessibility of the displayed immunogenic epitopes by using Ribosome Display [26], a powerful *in vitro* selection technique, to generate neutralizing antibodies *in vitro* from a naïve camelid single-domain antibody (nanobody) library. We demonstrate the prowess of our ADDoCoV design by immunizing mice, eliciting antigen-specific IgA and IgG antibody responses *in vivo,* notably also by intranasal (IN) vaccination, and through induction of mucosal immune responses, with the potential to induce stronger and more long-lasting indirect effects, especially in the context of a largely immune-primed global human population.

In parallel, we utilized the neutralizing nanobodies we had generated by Ribosome Display, to create an ADDomer-based multivalent superbinder nanoparticle which we called Gigabody, displaying multiple copies of the nanobody. To attach the nanobodies to the ADDomer scaffold, we mimic the mechanism that fiber proteins exploit in native adenovirus. Gigabody bound, with picomolar affinity, S proteins of SARS-CoV-2 including Omicron, an immune-evasive variant, and effectively abolished SARS-CoV-2 virion attachment to its cellular receptor, angiotensin-converting enzyme 2 (ACE2), demonstrating its potential for blocking SARS-CoV-2 infection reminiscent of the mechanism of monoclonal antibody treatments.

Taken together, we implement a novel, generic, integrated pipeline combining synthetic, computational and high-resolution structural methods with *in vitro* antibody selection and *in vivo* immunization to rapidly deliver, in parallel, reagents for active and passive immunization to combat respiratory diseases. Our pipeline approach provides a blueprint for the design of cost-effective, convenient to manufacture, easy-to-administer nanoparticle vaccines and multivalent, picomolar superbinders, for use in active and passive immunization, respectively, with the potential to prevent and combat infectious disease outbreaks also in resource-limited settings.

## RESULTS

### Self-assembling thermostable ADDomer-based COVID-19 candidate vaccine ADDoCoV

**Figure 1:**
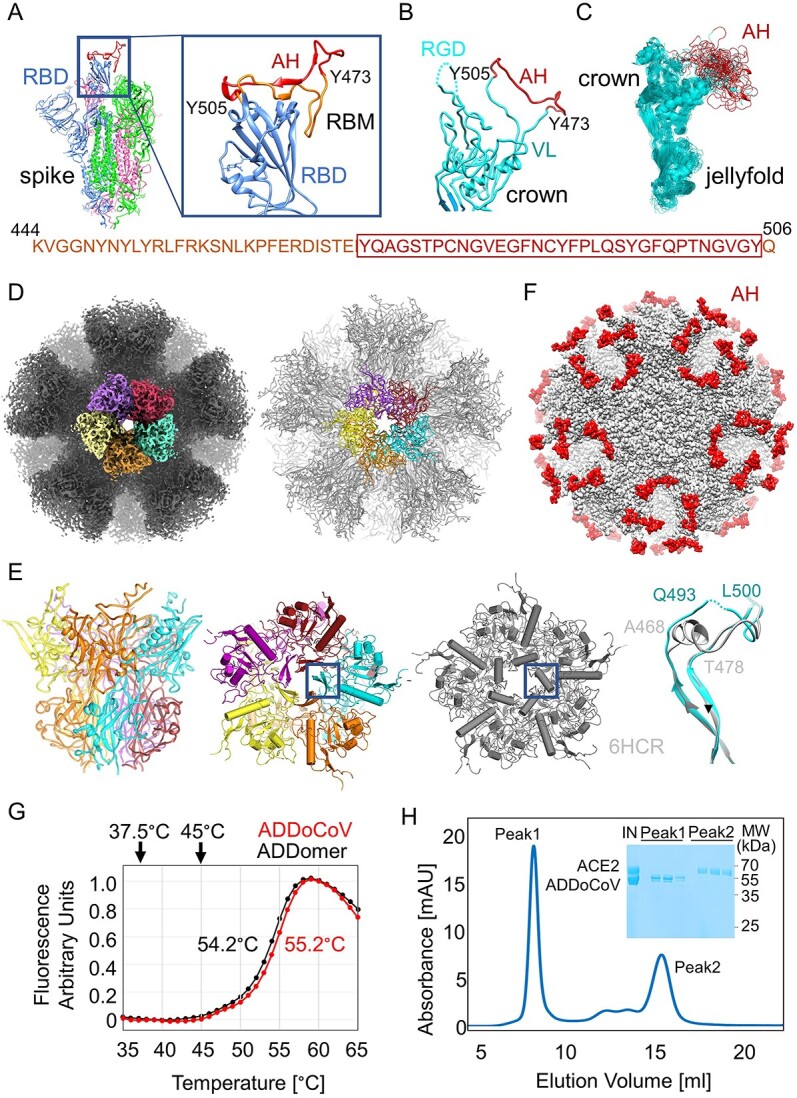
**Self-assembling thermostable ADDoCoV candidate vaccine.** (**A**) SARS-CoV-2 S protein (magenta, green and blue) shown in the open conformation (PDBID 7A94) [85]. The zoom-in (right) on the RBD in the “up” position (blue) depicts an ordered RBM (AA sequence provided below). The AH epitope (residue 473–505) is highlighted in red. (**B**) The ADDomer protomer crown domain (cyan) is shown. VL, variable loop; RGD, arginine-glycine-aspartate loop. AH (red) was inserted in VL. (**C**) MD simulations of the ADDoCoV protomer showing highly defined crown domain (cyan) while the AH epitope (red) in VL samples a range of conformations (RGD loop omitted for clarity). (**D**) Cryo-EM density (left) and model (right) of ADDoCoV. Five protomer (purple, firebrick, cyan, orange, yellow) form one penton. Twelve pentons form the nanoparticle. (**E**) One penton is depicted in a side view (left) and from the top (middle), colored as in panel d. For comparison, a penton from the ADDomer scaffold (PDBID 6HCR) [28] is shown (gray). A central region is boxed. Overlay of the boxed regions highlights unfolding of a central a-helix in the ADDoCoV protomer crown domain. (**F**) Model of ADDoCoV (gray) with AH epitopes colored in red. (**G**) Thermal unfolding curves of ADDomer scaffold (black) and ADDoCoV (red) demonstrate high thermotolerance. Melting temperatures are indicated. (**H**) SEC profile and Coomassie-stained SDS PAGE section (inset) showing ADDoCoV (Peak1) and ACE2 (Peak2) eluting separately. IN, injected sample.

The SARS-CoV-2 virion surface is decorated by S, a trimeric glycoprotein mediating cell attachment and infection [27–29]. Each S monomer contains a receptor binding domain (RBD) comprising the receptor binding motif (RBM). In the open form adopted by S, the RBM is positioned to interact tightly with ACE2 (Fig. 1A). Early in the pandemic, the sequence of SARS-CoV-2 S became available, when the genome of the original virus (WHO denomination “Ancestral strain”) became available. We used alignments with SARS-CoV S to delineate peptide regions in the RBM, which we could use as a putative antigenic epitope for genetic insertion into the protomer we had designed that forms the ADDomer nanoparticle [23]. An epitope (AH) encompassing 33 amino acids of the ancestral SARS-CoV-2 RBM spanning residues Y505 to Y473 inclusive (Fig. 1) was introduced into the insertion site of the variable loop (VL) of the protomer (Fig. 1B). The protomer adopts a bipartite structure (Fig. 1B,C), made up of a crown domain containing flexible loops and a jelly-fold domain mediating multimerization into pentons as well as the formation of the dodecahedron by establishing inter-penton contacts. Modeling by Rosetta design and MD simulations revealed AH conformational flexibility (Fig. 1C).

**Figure 2:**
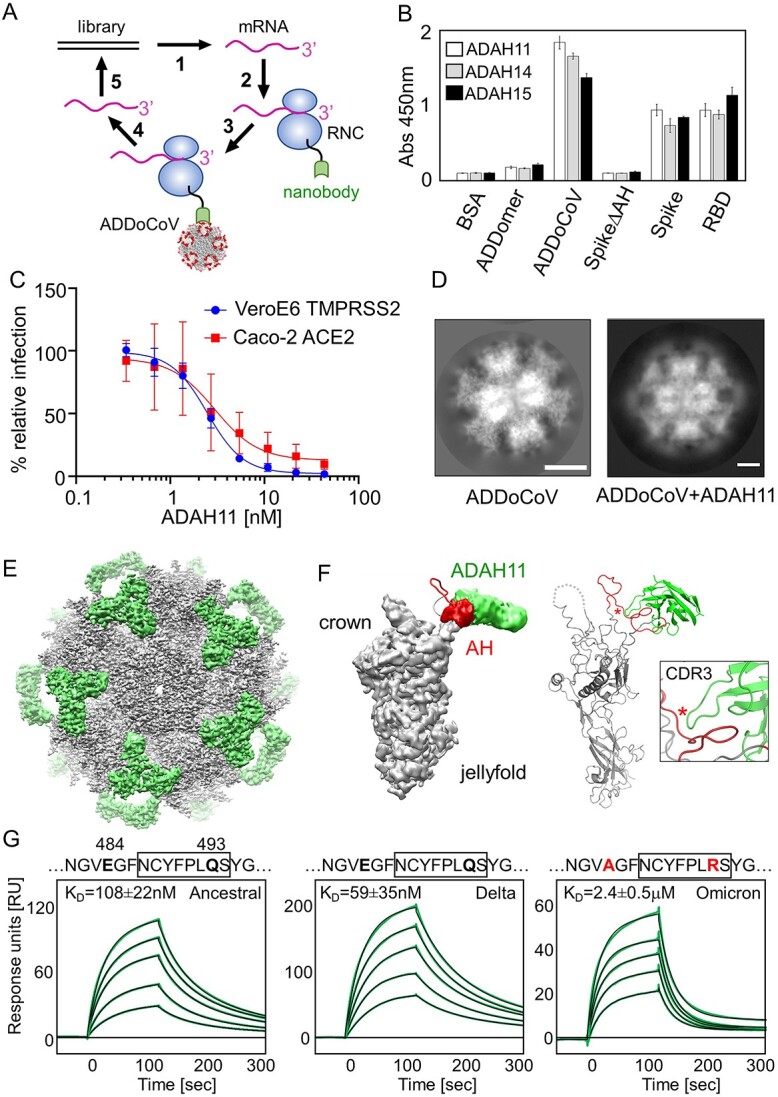
***In vitro* generated neutralizing nanobodies validate ADDoCoV design.** (**A**) Ribosome display in a schematic view. A DNA library encoding nanobodies is transcribed [1] and translated [2] *in vitro*. The stop codons in mRNAs are deleted. Resulting RNCs displaying nanobodies are used for panning [3] against ADDoCoV antigen. After washing, the mRNA of bound RNCs is eluted [4] and DNA recovered by RT-PCR [5]. The process is iterative. (**B**) ELISA of three selected nanobodies evidencing binding to ADDoCoV, S and RBD, but not to BSA, ADDomer scaffold, or a S mutant with AH deleted (SpikeΔAH). (**C**) SARS-CoV-2 neutralization by ADAH11 using ACE2 expressing Caco-2 (red) and TMPRSS2-expressing Vero E6 cells (blue). Dilutions were sampled in triplicates. EC50 values are 2.5 nM (VeroE6 TMPRSS2) and 3 nM (Caco2 ACE2), respectively. (**D**) Reference-free 2D class averages of ADDoCoV (left) and of ADDoCoV-ADAH11 nanobody complex (right) evidence halo of density corresponding to bound nanobody. (**E**) Cryo-EM structure of ADDoCoV (gray density) with bound nanobody (green). (**F**) Symmetry expansion (left) of ADDoCoV protomer (gray) with nanobody (green, filtered to ~10 Å resolution) bound to AH epitope (red). The corresponding molecular model (right) suggests ADAH11 recognizing a central section of AH. Location of an arginine residue in the ADAH11 CDR3 is marked (asterisk). (**G**) SPR of ADAH11 binding to immobilized Ancestral (left), Delta (middle) and Omicron (right) RBDs, at concentrations 50 to 250 nM for Ancestral and Delta, and 1 to 3 μM for Omicron. Epitope sequences are provided (top). Section bound by ADAH11 is boxed. Mutations in the Omicron RDB are highlighted (red). K_D_s are indicated.

AH containing protomer was produced following our established protocol [25] resulting in highly purified ADDoCoV adopting the dodecahedral structure characteristic of this protein nanoparticle (Fig. S1). We determined the cryo-EM structure of ADDoCoV at 2.36 Å resolution, providing near-atomic insights (Fig. 1D, Figs S2 and S3, Table S1, Movie S1). In a previous X-ray crystallographic study, a central α-helix had been identified, thought to stabilize the adenoviral penton by coordinating a bivalent ion, Ca^2+^, via glutamate residues [30]. In ADDoCoV, this α-helix seemingly underwent a helix-to-disorder transition and cation coordination was not observed (Fig. 1E). ADDoCoV contains 60 AH epitopes exposed on the nanoparticle surface in flexible loops, available for antibody binding (Fig. 1F). We probed the dynamics of ADDoCoV by MD simulations guided by the cryo-EM structure. For about a third of the simulated trajectory, the AH epitope adopted a conformation closely resembling the arrangement observed in the open form of SARS-CoV-2 S, with the RBD in the “up” position, positioned to engage ACE2 (Fig. 1E, Fig. S4).

A key feature of the self-assembling ADDomer scaffold resides in its thermostability [23]. We observed virtually identical melting temperatures (~55°C) of ADDomer and ADDoCoV, confirming that, irrespective of AH epitope insertion, the advantageous thermal feature is maintained (Fig. 1G) [23]. The AH epitopes displayed on the ADDoCoV nanoparticle comprise 33 amino acid residues of the SARS-CoV-2 RBM, which is itself about 60 residues long. We deliberately chose the shorter AH epitope for ADDoCoV to preclude potentially detrimental effects that could be caused by ADDoCoV sticking to cellular receptor ACE2. Size exclusion chromatography (SEC) of a mixture of ADDoCoV and highly purified, recombinant ACE2 showed no association with ACE2, notwithstanding the presence of multiple AH epitope copies on the ADDoCoV nanoparticle (Fig. 1H).

Moreover, we assessed the size distribution and zeta potential of ADDoCoV particles by dynamic light scattering (Fig. S5). The particles exhibited a monodisperse size distribution, evidenced by a polydispersity index (PDI) of 0.16. This suggests a uniform particle population, potentially leading to a favorable pharmacodynamic profile [31]. The zeta potential (measure for electrokinetic potential in colloidal dispersions) for ADDoCoV particles was determined to be −24.8 mV (±1.1 mV). Such a potential has been previously associated with enhanced cellular uptake and reduced aggregation in other nanoparticle systems [32].

In summary, we designed ADDoCoV comprising 60 copies of AH, a SARS-CoV-2 RBM derived epitope, determined ADDoCoV architecture and integrity at near atomic resolution, sampled the dynamics of the AH epitopes displayed on the ADDoCoV surface and demonstrated thermostability and uniformity of this COVID-19 nanoparticle vaccine candidate.

### *In vitro* generated SARS-CoV-2 neutralizing nanobody binders by Ribosome Display

The rationale for the ADDoCoV vaccine design is to elicit antibodies that can bind the RBM, and thus prevent SARS-CoV-2 attachment to ACE2, neutralizing the virus. A prerequisite for this is authenticity and accessibility of the AH epitope in the context of the ADDoCoV nanoparticle. To validate our design, we used Ribosome Display to select antibody binders from a naïve synthetic nanobody library, with ADDoCoV as an antigen (Fig. 2A). In Ribosome Display, a DNA library encoding for nanobodies is transcribed and translated *in vitro* [26]. In the library, the stop codons are deleted and replaced with a DNA sequence encoding an oligopeptide spacer. Thus, *in vitro* transcription and translation gives rise to ribosome nascent chain complexes (RNCs) coupling the genotype (mRNA) to the phenotype (nanobody), tethered to the ribosome. RNCs comprising specific nanobody binders are rapidly selected by panning on ADDoCoV immobilized on a surface. After washing away unbound RNCs, the remaining mRNA is recovered by dissociating the bound RNCs. Reverse transcription and PCR regenerates a DNA pool enriched for specific nanobody binders (Fig. 2A). By enzyme-linked immunosorbent assay (ELISA), we identified nanobodies that bound ADDoCoV as well as SARS-CoV-2 S and RBD, but not bovine serum albumin (BSA), native ADDomer scaffold, and S lacking the AH epitope (Fig. 2B, Table S2). A nanobody identified in this way, ADAH11, showed efficient virus neutralization in live SARS-CoV-2 infection assays using two different ACE2-expressing cell lines (Caco-2-ACE2 and VeroE6-TMPRSS2) (Fig. 2C).

ADAH11 was expressed and purified to homogeneity and tested for binding to highly purified ADDoCoV by SEC confirming complex formation (Fig. S6). Purified ADDoCoV-ADAH11 complex was analyzed by cryo-EM (Fig. 2D–F, Fig. S7, Table S3). Comparison of reference-free 2D class averages of ADDoCoV-ADAH11 or ADDoCoV, respectively, clearly indicated additional density for the nanobody containing complex (Fig. 2D). The cryo-EM structure of the ADDoCoV-ADAH11 complex revealed nanobody binding to the crown domains comprising the AH epitope (Fig. 2E,F, Fig. S7E). The arrangement of the pentons within the dodecahedron locates the AH epitopes in apparent triangles on the ADDoCoV surface. This is reflected by the triangular shape of the extra density stemming from nanobody binding (Fig. 2E). The limited resolution of the cryo-EM density in these more flexible outer regions only allowed rigid body docking of the nanobody, suggesting that ADAH11 is interacting with a central segment of the AH epitope in the protomer crown domain (Fig. 2F). Nanobody binding to cognate antigen is typically dictated by complementarity-determining region 3 (CDR3). In ADAH11, CDR3 comprises an arginine residue, R105 (Fig. 2F, Fig. S8, Table S2). By using surface plasmon resonance (SPR) by Biacore, we characterized ADAH11 binding to the SARS-CoV-2 S RBDs of the ancestral virus, as well as to variants of concern (VOCs) Delta and Omicron (Fig. 2G). ADAH11 bound immobilized S RBD of Ancestral and Delta, both, with similar, low nanomolar affinity (K_D_ of 108and 59 nM, respectively). Omicron S RBD, in contrast, was bound significantly less tightly (K_D_ of 2.4 μM). The epitope sequences juxtaposed to ADAH1 in the RBMs of Ancestral and Delta on one hand, and Omicron on the other, differ by a single Q to R mutation (Fig. 2G). We speculate that the presence of R105 in CDR3 of ADAH11 may contribute to the reduced binding to Omicron, possibly due to steric hindrance and/or charge repulsion; however, the limited resolution of the cryo-EM map precluded atomistic interpretation.

To summarize, a nanobody specific for an epitope derived from the RBM in the SARS-CoV-2 RBD was selected by Ribosome Display. This *in vitro* generated nanobody bound ADDoCoV, cross-reacted with the RBM in SARS-CoV-2 S, and neutralized live SARS-CoV-2 in cell-based infection assays, validating the authenticity and accessibility of the RBM-derived AH epitope displayed on the ADDoCoV nanoparticle vaccine.

### ADDomer-based ultrahigh-affinity Gigabody displaying SARS-CoV-2 nanobody binders

**Figure 3:**
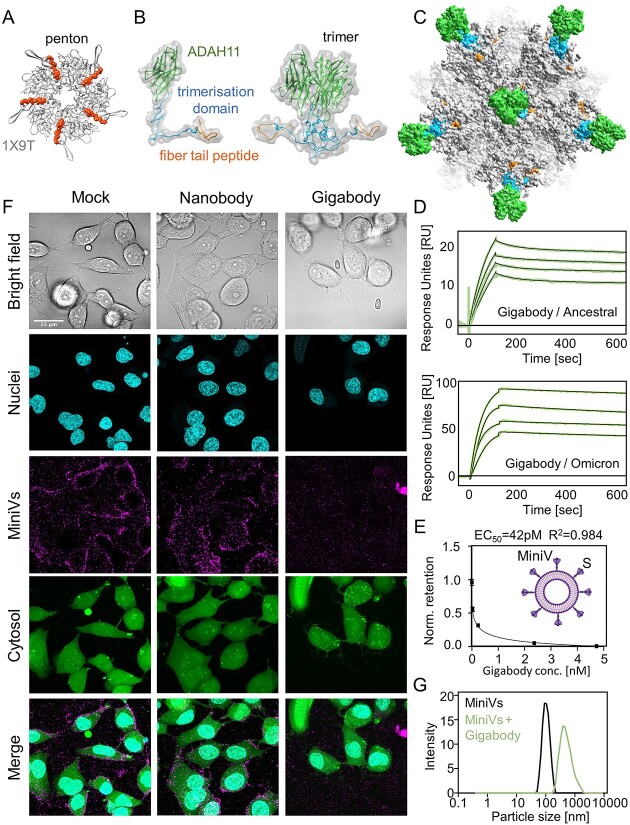
**Multivalent picomolar affinity Gigabody against SARS-CoV-2 RBM.** (**A**) Top view of an adenovirus penton base (gray) in complex with N-terminal fiber peptide (orange) is shown (PDBID 1X9T) [30]. (**B**) Protein engineering of ADAH11 nanobody (green) with a C-terminal T4 foldon trimerization domain (blue) and fiber tail peptide (orange) results in a trimeric complex (right). (**C**) Gigabody comprising 12 trimers bound via fiber tail peptides to pentons, displaying a total of 36 ADAH11 nanobodies. (**D**) SPR of Gigabody interactions with immobilized Ancestral (above) and Omicron (below) RBDs evidence very slow dissociation, consistent with tight (picomolar) binding. Gigabody concentrations of 0.5 to 2.0 nM (with immobilized Ancestral RBD) and of 1 to 2.5 nM (with immobilized Omicron RBD) were used. (**E**) Quantification of MiniV retention in ACE2-expressing A549 cell monolayers 2.5 h after incubation. Competitive binding of Gigabody to MiniV-presented S was assessed in a serial dilution series. Graph shows mean standard deviations from three technical replicates. IC50 is indicated. (**F**) Laser scanning confocal microscopy images of ACE2-expressing A549 cells 2.5 h after incubation with synthetic SARS-CoV-2 MiniVs decorated with S glycoprotein. MiniVs were either left untreated or exposed to 500 nM ADAH11 nanobodies or 1.6 nM Gigabody (corresponding to equal final protein concentration) for 30 min before addition to the cell cultures. Scale bar is 50 μm. (**G**) Dynamic light scattering analysis of Gigabody-mediated MiniV aggregation. MiniVs hydrodynamic size distribution is shown for untreated controls, and MiniVs that were pre-treated with 1.6 nM Gigabody for 30 min, respectively.

In adenovirus, the penton represents the base for attachment of the adenoviral fiber proteins that form characteristic protrusions at the vertices of the adenoviral capsid. The fiber adopts a trimer of three identical fiber proteins. Attachment to the penton base is mediated by a highly conserved, proline- and tyrosine-rich N-terminal fiber tail peptide present on each of the monomers (Table S4). The fiber tail peptide binds to a tailormade fiber tail peptide-binding cleft on the penton base. In a previously reported crystal structure of an adenoviral penton bound to isolated fiber tail oligopeptides, all five binding clefts were occupied (Fig. 3A) [30]. In the adenovirus, binding of the trimeric fiber to the penton base will result in two of five clefts remaining unoccupied.

The nanobodies we selected by Ribosome Display neutralized live SARS-CoV-2, most likely by blocking interactions with the ACE2 receptor due to steric hindrance. The nanobodies we obtained in this way were characterized by binding affinities of about 100 nM to their target antigen (Fig. 2G, Fig. S9). Multimerization of a nanobody can result in much tighter binding by increased avidity. We set out to exploit the principles of adenoviral fiber attachment using nanobody ADAH11 as a starting point, with the aim of creating an ultra-high affinity superbinder, “Gigabody”, displaying multiple copies of ADAH11, with the potential to forestall SARS-CoV-2 infection, which conceivably, could be utilized for passive immunization.

We had designed ADDoCoV based on a penton base protomer derived from human adenovirus serotype Ad3, which can efficiently self-assemble into a dodecahedron [23]. For Gigabody, we chose a different protomer, derived from chimpanzee adenovirus AdY25 to form the ADDomer scaffold (Table S5). Inspired by the adenovirus fiber, we designed a nanobody trimer by fusing a T4 phage derived trimerization domain (T4 foldon) preceded by the AdY25 fiber tail, to the N-terminus of ADAH11 (Fig. 3B, Table S6). Next, Gigabody was produced by mixing the trimers with AdY25-derived ADDomer, purified by SEC and dodecahedron formation confirmed by negative-stain EM (Fig. S10). Due to the 3:5 symmetry mismatch of ADAH11 trimer and penton, the trimer structure cannot be resolved at high resolution by cryo-EM, and computational modeling was used to illustrate the geometry of the Gigabody nanoparticles (Fig. 3C, Fig. S11, Movie S2). Fully occupied Gigabody presents 36 ADAH11 nanobodies arranged in 12 trimers, which should substantially increase binding to the cognate AH epitope by increasing avidity. We tested Gigabody binding to SARS-CoV-2 Ancestral S RBD by SPR. As expected, binding improved substantially, from ~100 nM for monomeric ADAH11 to picomolar for the Gigabody, driven by very slow dissociation (Fig. 3D). Moreover, with monomeric ADAH11, we had observed a significant drop in binding affinity from Ancestral S RBD (100 nM) to Omicron S RBD (2.4 μM) whereas Gigabody binding to Ancestral and Omicron was virtually identical, in the picomolar range (Fig. 3D). This indicates that Gigabody, by presenting multiple copies of ADAH11, rescues the comparatively low, micromolar affinity binding by the nanobody to Omicron S RBD due to avidity (Fig. 3D).

We tested the capacity of Gigabody to abrogate virion attachment to ACE2 expressing cells. We used synthetic minimal SARS-CoV-2 virions decorated with highly purified S glycoproteins (SARS-CoV-2 MiniVs) as a model system, affording complete control of experimental parameters. We had used synthetic MiniVs previously to reveal fatty-acid coupled adaptive immunogenicity of SARS-CoV-2 [33]. SARS-CoV-2 MiniVs faithfully recapitulate viral attachment and can be studied in a regular laboratory setting (biosafety level 1), in contrast to live virus. We assessed competitive binding of Gigabody to MiniV-presented S in a serial dilution (Fig. 3E) and analyzed attachment of SARS-CoV-2 MiniVs to ACE2-expressing A549 cells exposed to Gigabody by laser scanning confocal microscopy (Fig. 3F). We observed quantitative inhibition of SARS-CoV-2 MiniV cell attachment at a Gigabody concentration of 1.6 nM (Fig. 3F). Previously, we had determined the half maximal effective concentration (EC50) of ADAH11 nanobody in terms of MiniV retention as 117 nM [33]. Gigabody EC_50_ is 42 pM (Fig. 1E), which is 300-fold lower, closely mirroring the respective binding properties of single nanobody and Gigabody, respectively, in SPR measurements (Fig. 2G, Fig 3D). Due to the presence of multiple nanobody trimers, Gigabody could in theory induce virion aggregation, similar to agglutination. We analyzed the hydrodynamic size distribution of SARS-CoV-2 MiniVs by dynamic light scattering, confirming aggregation following Gigabody addition, with particle sizes increased to ~1000 nm from the diameter of a single virion (~100 nm) (Fig. 3G). Furthermore, we measured the size distribution and zeta potential of the gigabody particles by dynamic light scattering (Fig S5). The zeta potential was measured at −15.3 mV (±1.2 mV), a value anticipated to inhibit aggregation. In addition, the particles exhibited a monodispersed size distribution with a PDI of 0.14.

Taken together, we have mimicked the design of the adenoviral fiber, and its attachment mechanism in the adenovirus, to generate a Gigabody nanoparticle decorated with multiple copies of trimerized ADAH11. By avidity, Gigabody binds the cognate target in the RBM of SARS-CoV-2 S with greatly enhanced, picomolar affinity as compared with nanomolar binding by ADAH11 nanobody alone. Moreover, Gigabody binding to the RBMs in Ancestral S and Omicron S is virtually identical, while ADAH11 alone binds Omicron with significantly reduced affinity as compared with Ancestral, presumably due to mutations accrued by Omicron in the RBM. Finally, Gigabody effectively abolishes attachment of SARS-CoV-2 MiniVs in cell-based assays and can mediate virion agglutination. Intriguingly, Gigabody thus may represent an attractive avenue for passive immunization, based on the same nanoparticle scaffold concept, ADDomer, that we used for ADDoCoV, exploiting assembly principles of the adenovirus from which ADDomer is derived, and utilizing antibody binders generated *in vitro* against ADDoCoV used as an antigen.

### ADDoCoV immunization experiments *in vivo* in mice

**Figure 4:**
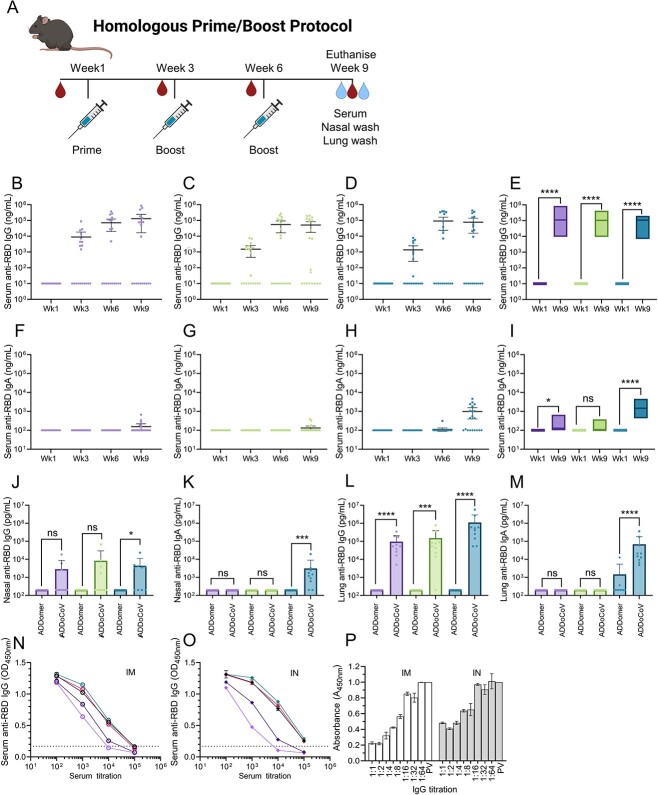
**ADDoCoV *in vivo* immunization elucidates nasal vaccination route.** (**A**) Schematic of immunization schedule and end point. (**B–E**) Determination of anti-RBD specific IgG binding antibodies induced through sub-cutaneous immunization (purple), intra-muscular immunization (green) and intra-nasal immunization (blue). (**F–I**) Comparison of anti-RBD specific IgA binding antibodies induced through SC immunization, IM immunization and IN immunization. (**J**,**K**) Determination of anti-RBD specific IgG binding antibodies in NW induced by IM or IN immunization. (**L**,**M**) Determination of anti-RBD-specific IgG binding antibodies in lung washes induced by IM or IN immunization. Control unvaccinated animals are represented by □, vaccinated animals by ■ symbols. Statistical analysis utilized two-sided Mann–Whitney tests. (**N**,**O**) Assessment of cross-variant binding for total IgG antibodies induced through IM (n) or IN (o) immunization. Black (Alpha variant), pink (Delta), teal (Ancestral), purple (Beta), mauve (Omicron). Dotted line represents upper 99% CI of blank controls. (**P**) sVNT [33] assessing total IgG antibodies induced through IM (white bars) or IN (gray bars) immunization, normalized to pre-vaccination sample (PV) included as control (bars far right). Dilutions indicated were sampled in triplicates.

Traditional routes of vaccine administration, such as intramuscular (IM), subcutaneous (SC) and intradermal vaccination, generally induce significant concentrations of antigen-specific IgG that are detectable in the recipient’s serum. In the current study, we compared traditional IM or SC vaccination with IN vaccination as this route may also induce strong systemic responses as well as significant levels of antigen-specific antibody detectable in mucosal secretions. We hypothesized that IN vaccination might be beneficial for a SARS-CoV-2 vaccine, as it could increase front-line mucosal defenses against a pathogen that infects the respiratory tract and thus impact on viral transmission more effectively than the vaccines currently in use. Therefore, we tested the immunogenicity of ADDoCoV using a homologous prime-boost protocol (Fig. 4A). Specifically, we tested the immunogenicity of ADDoCoV in mice as compared with the naïve ADDomer nanoparticle as a control, when administered via SC, IM and IN routes. As shown (Fig. 4B–D), after the vaccine prime, the ADDoCoV formulation resulted in 100% seroconversion (*n* = 10 mice/group) regardless of the route of administration. Elevated concentrations of anti-RBD specific serum immunoglobulin-G (IgG) were detected during study weeks 3, 6 and 9. When compared with baseline, anti-RBD IgG antibody titres in serum in week 9 after vaccination were significantly increased across all conditions (^*^^*^^*^^*^P ≤ 0.0005) (Fig. 4E).

Given the important role that immunoglobulin A (IgA) plays in defense against mucosal pathogens [34], we also measured the serum anti-RBD IgA response after ADDomer and ADDoCoV vaccination. Detectable anti-RBD IgA was also induced in serum (Fig. 4F–H), however the IgA response developed more slowly than the IgG response, with anti-RBD IgA antibody only being significantly elevated at week 9 in the SC and IN groups compared with the week 1 baseline control (Fig. 4I). Importantly, only the IN group showed 100% seroconversion after ADDoCoV administration, with the IN route also resulting in the highest anti-RBD IgA response in serum. Taken together, these data show that the ADDoCoV vaccine is immunogenic and elicits antigen-specific antibody responses in the serum of vaccine recipients, irrespective of the route of administration. However, the magnitude, kinetics and isotype of the elicited antibody response are influenced by the route of administration.

To gain a more in-depth understanding of the elicited antibody response after SC, IM and IN vaccination with ADDoCoV at the end of the study, we collected nasal (Fig. 4J,K) and lung washes (Fig. 4L,M) to quantify the anti-RBD IgG and IgA responses in mucosal secretions. As expected, systemic routes of vaccination failed to induce detectable anti-RBD IgG and IgA responses in nasal secretions with only the IN administration of ADDoCoV resulting in significantly elevated concentrations of IgG (^*^P ≤ 0.05) and IgA (^*^^*^^*^P ≤ 0.0005) compared with the ADDomer control vaccine. Interestingly, a distinct pattern of anti-RBD antibody induction was observed in lung washes. Here, we found that significant levels of anti-RBD IgG were induced regardless of the route of vaccine administration. However, despite the IM, SC and IN groups all having significant levels of anti-RBD in lung washes, it should be noted that the IN group had the highest. Interestingly, only in the IN group were significant levels of anti-RBD IgA induced in lung washes (Fig. 4M). Taken together these results suggest that IN immunization may induce mucosal antibody responses more efficiently than systemic routes of administration and support the use of this route of administration to enhance interruption of acquisition and onward transmission of infection.

Next, we sought to evaluate whether the antibodies induced by ADDoCoV vaccination were cross-reactive against a range of clinically relevant SARS-CoV-2 variants (Ancestral, Alpha, Beta, Delta and Omicron), which would be important for broad protection, given the rapid and continuing evolution and diversification of SARS-CoV-2 in the human population. IgG antibodies induced following both IM and IN administration of the vaccine bound all SARS-CoV-2 S RBDs tested, with close to identical binding observed to Ancestral, Alpha and Delta RBDs, and reduced binding observed for Beta and Omicron RBDs (Fig. 4N,O). Of note, a reduction in binding to Omicron RBD, as compared with Ancestral and Delta RBDs, was also observed for ADAH11, the nanobody generated by Ribosome Display (Fig. 2G), suggesting that our *in vitro* selection may reproduce some of the antibody binding characteristics likewise occurring *in vivo*.

**Figure 5:**
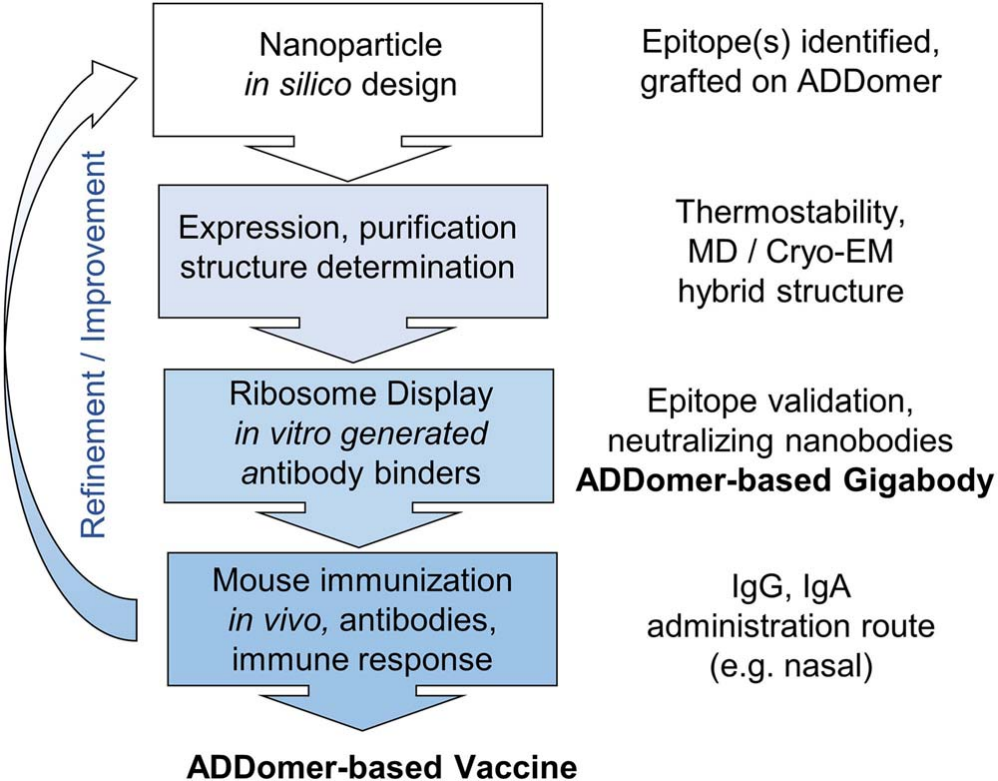
**Integrated pipeline for ADDomer-based nanoparticle vaccine and Gigabody**. Integrating *in silico* design, cryo-EM, MD simulations, *in vitro* selection by Ribosome Display, synthetic SARS-CoV-2 virions and live virus neutralization, and validation in an animal model, for generating ADDomer-based nanoparticle therapeutics for active (vaccine) and passive (Gigabody) immunization. With established protocols for each step, the process from immunogenic epitope identification and grafting onto ADDomer, until release to animal studies, is rapid and can be completed in about 5 weeks. The process can be repeated iteratively to refine and optimize, for instance by including diverse additional B and T epitopes in the design. In parallel, multivalent Gigabody superbinder is delivered in the process.

We showed that the ADDoCoV vaccine is immunogenic and induces antibodies in serum and mucosal secretions, which are cross reactive against diverse SARS-CoV-2 S protein variant RBDs. Next, we used serum IgGs to perform a surrogate virus neutralization assay (sVNT) [35], based on competition with Ancestral RBD bound to immobilized ACE2. We observed moderate neutralizing activity in our tests (Fig. 4P). Antibody-mediated neutralization is thought to be important in protection against infection by SARS-CoV-2 [36]. At the same time, absence of strong neutralization does not equate to lack of protection by serum antibodies, as even entirely non-neutralizing antibodies, which bind target proteins in pathogens specifically, can confer strong protection through antibody-dependent cellular functions [37–39]. We note that our *in vitro* generated nanobody binder, ADAH11, showed strong viral neutralization (Fig. 2C). The serum antibodies, whilst specifically binding S RBM, may be characterized by moderate binding affinity, restricting their performance in the sVNT assay. It is conceivable that iterative adjustment and refinement of the vaccine design (Fig. 5), for instance by incorporating additional epitopes, could result in stronger binding and more efficient neutralization by the antibodies generated.

## DISCUSSION

We present here our integrated approach combining synthetic, computational and high-resolution structural methods with *in vitro* selection and *in vivo* immunization techniques (Fig. 5), to design, produce and validate nature-inspired nanoparticle-based reagents to combat infectious pathogens. Utilizing this pipeline, we developed ADDoCoV, a nanoparticle vaccine that can be applied nasally, a route unparalleled in ease of administration. Moreover, we crafted Gigabody, a novel multivalent superbinder, against SARS-CoV-2 including immune-evasive variants. Both vaccine and Gigabody have in common that they rely on the same synthetic self-assembling ADDomer scaffold concept.

Our ADDoCoV vaccine displays 60 copies of an epitope derived from the RBM of SARS-CoV-2, mediating attachment to its receptor, ACE2. We used *in vitro* selection of neutralizing nanobodies to confirm authenticity and accessibility of the epitope displayed, followed by *in vivo* immunization studies in mice to characterize the immune response and how this is influenced by route of administration. Our mouse experiments elucidated nasal administration as a viable route for ADDoCoV vaccination, eliciting specific serum IgG and mucosal IgA responses *in vivo*. We note that our *in vitro* selection by Ribosome Display generated nanobody binders that exhibited properties similar to the antibodies in the sera of immunized mice. This point is relevant in the context of the principle of 3Rs (reduce, replace, refine) to limit animal use for biomedical research, here for prescreening suitable vaccine candidates with the desired properties regarding the antigen displayed.

In parallel, making use of the neutralizing nanobodies we selected *in vitro*, we created Gigabody, a multivalent superbinder nanoparticle, enabled by a previously unexplored docking feature of the ADDomer scaffold. Gigabody displays multiple trimers of the neutralizing nanobody, effectively blocks virion attachment to ACE2 expressing cells and could likely be administered similar to ADDoCoV via the nasal route, for passive immunization. We used ADDomer scaffolds formed by protomers of different origin—human Ad3 for vaccine and chimpanzee AdY25 for Gigabody, respectively, to minimize any risk of anti-platform immunity, if active and passive immunization steps were combined in a given treatment regimen, for potential synergistic effect. In this context, it is noteworthy that several adenovirus serotypes have been identified comprising penton base proteins that can adopt dodecahedrons, and thus could be conveniently used as further alternatives, for interventions against different infectious pathogens [24].

In our mouse immunization experiments, administration of ADDoCoV was sufficient to induce serum anti-RBD IgG production by SC, IM and IN routes, demonstrating that ADDoCoV is capable of inducing systemic immune responses to the relevant target. Since the ADDomer has been shown to self-adjuvant [23], adjuvant was not included in our experiments. Analysis of anti-RBD IgA responses at each time point revealed that protocol design, time and route of administration were important determinants of IgA induction. Serum anti-RBD IgA was detectable in peripheral blood samples only at week 9, following a complete prime-boost-boost protocol, showing a difference in seroconversion between IgG and IgA. Our results are consistent with maturation of the immune response following initial prime and subsequent boost treatments. Significant increases in serum anti-RBD IgA was observed only following SC or IN administration, while no significant increase in IgA production above baseline (treatment naïve) levels was detected in the IM treatment group. This is interesting given that mucosal immunity is thought to be a critical aspect of SARS-CoV-2 infection [40], and supports that the recruitment of the appropriate physiological, e.g. mucosal, responses is enhanced by appropriate delivery of vaccine to relevant tissues.

The prevalence of antigen specific (anti-RBD) local/mucosal antibody production was assessed in nasal washes (NW) and bronchoalveolar lavage (BAL) samples collected at week 9. Detection of vaccine induced local nasal IgG and IgA was limited to mice treated via IN ADDoCoV administration, whereas treatment via SC or IM routes was inefficient in the generation of nasal mucosal antibody, as no increase in anti-RBD IgG or IgA was detectable above control levels. In contrast, lung mucosal IgG production was not dependent on the route of vaccine administration and was detected in ADDoCoV treated mice via all treatment routes. Induction of lung mucosal anti-RBD IgA production remained limited by the route of vaccine delivery and was only detected in mice that received IN ADDoCoV administration. This is noteworthy as it further demonstrates specificity of induced mucosal responses according to route of administration, which was suggested by the presence of IgA in the serum of mice only after IN treatment. IgA is known to play a crucial role in the immune defense of mucosal surfaces, the first point of entry of SARS-CoV-2 [41].

The ADDomer scaffold comprises altogether three insertion sites per protomer [23], only one of which is currently occupied by AH in ADDoCoV. Cellular immunity mediated by T cells is known to play an important role in the protection against viral infection, mediating effective viral clearance, elimination of virus-infected cells, and long-term disease protection. ADDomer was shown to drain to lymph nodes and is efficiently taken up by antigen presenting cells [23]. Successful presentation of T cell epitopes, in addition to B cell epitopes, by ADDomer has been demonstrated recently for Type O foot-and-mouth disease virus, resulting in protective responses against the viral pathogen [42]. Although the epitope used for ADDoCoV was not designed to elicit a T cell response, there may be activation of other innate immune cells, and it would be illustrative to examine the broader immune response spectrum in future experiments to fully understand the efficacy of ADDomer-based nanoparticle vaccines. Moreover, a range of T cell epitopes in the SARS-CoV-2 proteome have been identified, and it is likely that by expanding our ADDoCoV design to include validated SARS-CoV-2 T epitopes in the currently unoccupied insertion sites in the ADDomer scaffold, a T cell response can also be induced against SARS-CoV-2. SARS-CoV-2 VOCs, in particular Omicron, are characterized by multiple mutations, increasingly evading existing antibody responses, requiring updated versions of current vaccines to confer immunity. We note that in our experiments, we observed reduced binding of both, serum antibodies and ADAH11 nanobody, to Omicron, which is dominating world-wide at the time of this report. Clearly, vaccines will need to be redesigned accordingly to address this. The ease of epitope insertion, and epitope alteration, on the genetic level renders the ADDomer scaffold particularly attractive for rapid, rolling update. We note that insertion of strings comprising several immunogenic epitopes in a row has been demonstrated for ADDomer [23]. We propose rapidly updated ADDoCoV nanoparticles, comprising strings of the respective B and T epitopes, as attractive candidates for recurring vaccination against SARS-CoV-2 VOCs. Again, to avoid pre-immunity issues, scaffolds of different origin could be used for booster vaccinations.

Affordable production is a key prerequisite for broad vaccine distribution in resource-limited settings. Other nanoparticle-based SARS-CoV-2 vaccine candidates are often made up of one or several different S proteins, or their RBDs, which are coupled to diverse scaffolds [43–45]. For instance, a SARS-CoV-2 mosaic vaccine comprised eight different S RBDs coupled to a separate nanoparticle [41]. A different nanoparticle vaccine candidate displays S on a nanoparticle scaffold, which itself is made up of different components, each produced in a different heterologous system [44]. In these nanoparticle vaccines all components, S, RBD and scaffolds, need to be produced and purified separately, then combined and repurified, multiplying manufacturing runs and associated costs. In contrast, ADDoCoV relies on genetically encoded multiepitope display by a single, one-component particle, requiring one production run only using established manufacturing technology, significantly reducing costs, and maintaining thermostability. Given that nanobodies are efficiently expressed intracellularly [46], we anticipate that Gigabody superbinders can likewise be produced in a single production run co-expressing nanobody trimers and the ADDomer scaffold simultaneously, reducing manufacturing costs.

The COVID-19 outbreak painfully reminds us of the critical importance of pandemic preparedness, requiring sophisticated approaches to facilitate and accelerate reagent development against emerging threats. The integrated pipeline we established enables rapid generation of ADDomer-based nanoparticle vaccines and multivalent Gigabody superbinders, both exploiting the same thermostable scaffold, for active and passive immunization, respectively. We demonstrate our approach here using SARS-CoV-2 as an example, but it could be applied as well for any other pathogen causing infectious disease, present and future.

## MATERIALS AND METHODS

### Protein production

#### ADDoCoV preparation

ADDoCoV was designed during the early pandemic before SARS-CoV-2 S structures became available, based on sequence comparison of the RBDs of SARS-CoV S, MERS-CoV S and SARS-CoV-2 S, and the structure of SARS-CoV bound to ACE2 receptor or neutralizing antibodies, respectively [47–49]. Variations of the oligopeptide sequence corresponding to the ACE2 RBM were then inserted into the ADDomer scaffold as described previously [23] and expressions carried out using the MultiBac baculovirus expression system following established protocols [50]. ADDoCoV, the candidate here described comprises a 33 amino acid sequence (AH epitope) in the VL insertion site (Fig. 1A, Table S1).

ADDoCoV purification was adapted from a previously established protocol [51]. Briefly, pellets were resuspended in Resuspension Buffer (50 mM Tris pH 7.5, 150 mM NaCl, 2 mM MgCl_2_ Buffer, 1 mL per 2.5 × 10^7^ cells) supplemented by EDTA-free complete protease inhibitor (Roche). Lysate was prepared by three cycles of freeze-thawing, cleared by centrifugation (40 000 g, 30 min), supplemented with Benzonase (Sigma-Aldrich) and incubated on ice for 2 h. Precipitate was removed by centrifugation (4000 g, 15 min), the supernatant passed through a 0.45 μm filter and subjected to SEC using a XK 26/100 column (GE Healthcare). Fractions containing ADDoCoV were pooled and further purified by ion exchange chromatography (IEX) using a 5 mL Bio-Scale Mini Macro Prep High Q (Bio-Rad) equilibrated in Buffer A (50 mM Tris pH 7.5, 150 mM NaCl) and a linear salt gradient from 0.15 M to 1 M NaCl. Highly purified ADDoCoV eluted at ~300 to 400 mM NaCl. Fractions were pooled and stored at ambient temperature or refrigerated (4°C). For animal studies, ADDoCoV particles were filtered through a 0.22 μm filter and further purified utilizing Detoxi-Gel (ThermoFisher Scientific) to remove endotoxins and dialyzed against PBS.

#### Receptor-binding domains

Biotinylated SARS-CoV-2 RBDs of Ancestral, Alpha, Beta, Delta and Omicron (B.1.1.529) were expressed and purified as described [52].

#### ADAH11 expression and purification

A synthetic gene (Genscript) encoding for ADAH11 was inserted into plasmid pHEN6 [53] resulting in construct pHEN6-ADAH11 comprising a PelB secretion signal at the N-terminus and a hexa-histidine and triple-FLAG tag on the C-terminus. Nanobody was expressed in *Escherichia coli* (*E. coli*) TG1 cells cultured in 2xYT media, induced with 1 mM Isopropylthio-β-galactoside (IPTG) overnight (~16 h) at 30°C, harvested by centrifugation and pellets stored at −80°C. Cell pellets were resuspended in ice cold TES (50 mM TRIS pH 8.0, 20% w/v sucrose, 1 mM EDTA) and incubated for 1 h at 4°C. Next, Shock Buffer (20 mM Tris pH 8.0, 5 mM MgCl_2_) was added followed by incubation for 1 h at 4°C. Supernatant was cleared by centrifugation, applied to 1 mL HisPur Ni-NTA resin (ThermoFisher Scientific), and incubated for 1 h at 4°C with agitation. After washing with Wash Buffer (50 mM HEPES pH 8.0, 200 mM KCl, 10 mM Imidazole), ADAH11 was eluted with Elution Buffer (150 mM imidazole 50 mM HEPES pH 8.0, 200 mM KCl). Fractions containing ADAH11 protein were pooled, dialysed into PBS, and further purified by SEC using a S200 10/30 GL column (Cytiva) equilibrated in PBS. Eluted ADAH11 was concentrated to 1 mg/mL and stored at −80°C.

#### Gigabody preparation

ADDomer derived from chimpanzee adenovirus AdY25 comprising an A57S mutation (Table S1) was expressed and purified as described above for ADDoCoV. Following SEC and IEX, samples were sterile filtered, flash frozen in liquid nitrogen and stored at −80°C. The integrity of the final sample was confirmed using both reducing SDS-PAGE and negative stain EM.

A codon optimized synthetic DNA encoding fiber tail peptide (Table S5), T4-foldon trimerization domain and ADAH11 spaced by glycine-serine linker sequences (Table S6) was inserted into the pHEN6 plasmid (Genscript), expressed in T7 Express *E. coli* cells (New England Biolabs) cultured in Terrific broth medium and induced with 1 mM IPTG for overnight expression at 16°C. Cells were harvested by centrifugation (4000 g for 10 min), resuspended in Lysis Buffer (50 mM Tris–HCl pH 8, 300 mM NaCl, 10 mM Imidazole, 0.5 mg/mL Lysozyme), frozen at −20°C and thawed at 37°C for 10 min, followed by DNase treatment at 4°C (15 min). DNase treated sample was sonicated at 50% amplitude 4 times for 30 s, Pulse 1 s/1 s using Vibracell VC 750 (Sonics and Materials) and clarified by centrifugation (12 000 g for 15 min). Cleared supernatant was loaded onto a 5 mL Histrap FF crude Ni-NTA affinity column (Cytiva), washed with Wash Buffer (50 mM Tris–HCl pH 8, 300 mM NaCl, 50 mM Imidazole), and nanobody trimers eluted with Elution Buffer (50 mM Tris–HCl pH 8, 300 mM NaCl, 250 mM Imidazole). Elution fractions were pooled and concentrated to 500 μL using a 10 kDa MWCO Amicon centrifugal filter unit (EMD Millipore), and further purified by SEC using a Superose 6 HR 10/30 column (Cytiva) equilibrated with PBS. Peak fractions were pooled, aliquoted and stored at 4°C.

Gigabody was assembled by mixing purified ADAH11-Trimer and AdY25 A61S ADDomer in PBS at a molar ratio of 1:1.2 pentons to ADAH11-Trimer. After 1-h incubation rotating at 4°C, the mixture was subjected to SEC on a Superdex 200 10/300 GL column (GE Healthcare) equilibrated in PBS. Peak fractions containing Gigabody were pooled, concentrated using a 100 kDa MWCO Amicon centrifugal filter unit (EMD Millipore) and used fresh, or flash-frozen in liquid N_2_ for storage at −80°C.

### Thermostability measurements

Thermal shift experiments were performed using a ThermoFluor assay as described previously [23].

### Dynamic light scattering analysis of ADDoCoV and gigabody particles

AddoCoV and Gigabody size distribution and zeta potential was measured by dynamic light scattering with a Malvern Zetasizer Nano ZS system at final protein concentration of 300 μg/mL in PBS. Temperature equilibration time was set to 300 s at 25°C, followed by three repeated measurements for each sample at a scattering angle of 173° using the built-in automatic run-number selection. The material refractive index was set to 1.45 and solvent properties to *η* = 0.8882, *n* = 1.33 and *ε* = 79.0.

### Negative-stain sample preparation and electron microscopy

#### ADDoCoV

4 μL of 0.1 mg/mL ADDoCoV protein sample dialyzed into 25 mM HEPES pH 7.5, 150 mM NaCl, 2 mM EDTA was applied onto a freshly glow discharged (1 min at 10 mA) CF300-Cu grid (Electron Microscopy Sciences), incubated for 1 min, and manually blotted. 4 μL of 3% Uranyl Acetate was applied onto the same grid and incubated for 1 min before the solution was blotted off. Images were acquired at a nominal magnification of 49 000× on a FEI Tecnai 12 120 kV BioTwin Spirit microscope equipped with an Eagle 4 k × 4 k CCD camera.

#### ADDoCoV-ADAH11 complex

5 μL of 0.1 mg/mL ADDoCoV-ADAH11 complex sample was prepared as above. Images were recorded at 62 000× magnification corresponding to a pixel size of 1.63 Å/pix. A total of 5025 particles from 498 images were picked and reference free two-dimensional classification was performed leading to 1396 particles included in final 2D class averages (Fig. S7).

### Cryo-EM sample preparation and data collection

#### ADDoCoV

4 μL purified ADDoCoV (0.54 mg/mL) was applied to glow-discharged holey Quantifoil R 1.2/1.3 holey carbon grids (Agar Scientific), blotted for 2 s at 100% relative humidity and 4°C inside a Vitrobot Mark III, before plunge-freezing in 37% ethane-propane at liquid nitrogen temperature. Cryo-EM data were collected at 200 kV with a FEI Talos Arctica microscope equipped with a Gatan K2 direct electron detector and an energy filter at 20 eV slit width, using automated acquisition software (EPU). A total of 1375 dose-fractionated movies each containing 40 frames (0.2 s per frame) with an accumulated total dose of 44 e−/Å^2^ were recorded in counted super-resolution mode at a nominal magnification of 130 000× corresponding to a physical pixel size of 1.05 Å and a virtual pixel size of 0.525 Å using a defocus range of −0.7 to −2.2 μm (Figs S2 and S3, Table S2).

#### ADDoCoV-ADAH11 complex

3 μL of 1.2 mg/mL ADDoCoV-ADAH11 complex was loaded onto a glow discharged Quantifoil R1.2/1.3 holey carbon grid (Agar Scientific). The sample was incubated for 30 s at 90% relative humidity and 16°C inside Leica EM ACE 600 (Leica EM GP2 plunge freezer), blotted for 1.2 s and vitrified in liquid ethane at liquid nitrogen temperature. Data were acquired on a FEI Talos Arctica as described above. Data were collected in counted super-resolution mode at a nominal magnification of 130 000× with a physical pixel size of 1.05 Å/pix and a virtual pixel size of 0.525 Å/pix. The total dose of 55.6 e/Å^2^. Each movie was fractionated in 45 frames of 200 ms. A total of 11 800 micrographs were collected with a defocus range comprised between −0.8 and −2.0 μm.

### Cryo-EM data processing

#### ADDoCoV

Image processing was performed with the RELION 3.1 software package [54]. The micrographs were motion corrected using MotionCor2 [55] and contrast transfer function (CTF) information determined using gctffind4.1 [56]. A total of 1375 micrographs with CTF rings extending beyond 4 Å were selected for further processing. A total of 96 456 particles were boxed using RELION auto-picking software. 2D classification (Fig. S2) and 3D classification with imposed icosahedral symmetry was performed, followed by initial 3D-autorefinement. Further rounds of 3D-classification/refinement were carried out on 32 227 polished particles after CTF refinement and spherical aberration correction before using post-processing for masking and automatic B-factor sharpening. The resolution of the final map was determined to be 2.36 Å based on the Fourier Shell Correlation (FSC) = 0.143 criterion (Fig. S3). Local resolution was calculated using local resolution estimation program in RELION (Fig. S3B). 3D classification was performed using public cloud resources provided by Oracle Cloud Infrastructure as described previously [23].

#### ADDoCoV-ADAH11 complex

A total of 11 283 dose-fractionated movies were image processed as described above. A total of 68 258 particles were automatically picked using Relion 4.0 [57]. After three rounds of 2D classification, a total of 46 223 particles were selected for further 3D classification. The initial 3D model was filtered to 60 Å during 3D classification using eight classes. The best class of 13 950 particles was selected for the following 3D-autorefinement leading to a reconstruction of ~4.35 Å resolution. Subsequently, the maps were subjected to local defocus correction and Bayesian particle polishing in Relion 4.0. Global resolution and B-factor (−79.66 Å^2^) of the maps were estimated by applying a soft mask around the protein density using the gold-standard FSC criterion 0.143, resulting in an overall resolution of 4.06 Å (Fig. S7). Local resolution maps were generated using Relion 4.0. The refined particles stack was expanded 60-fold according to icosahedral symmetry. The symmetry expanded particle stack was then used as input for the masked 3D classification with the focus mask corresponding to one penton base protein and the ADAH-11 region created in UCSF Chimera [58]. The masked 3D classification was performed with eight classes resulting three good classes with densities for ADAH11 and penton base protein (Fig. 2E,F, Fig. S7).

### Cryo-EM model building and analysis

Homology modeling was performed using iTasser [59] starting from the human ADDomer structure (PDB ID 6HCR) [23]. Using COOT [60], the model was fitted manually into the EM map, followed by iterative positional and B-factor refinement using Phenix Real-Space software [61]. After adjustments in COOT, the model was evaluated using Molprobity [62].

### *In vitro* selection of specific nanobodies by Ribosome Display

#### Synthetic VHH nanobody library

For selection by using Ribosome Display, a synthetic nanobody library was constructed based on llama VHH subtypes IGHV1S1-S5 [63]. A consensus scaffold was designed with highest sequence identity to subtype1 (92%), taking into account available data regarding stability and expression yields of the different VHH subtypes in *E. coli*. Diversity was introduced in the CDRs by designing each position of CDR1 and CDR2 to comprise a set of amino acids that closely recapitulates natural diversity while reducing the presence of hydrophobic residues to avoid aggregation propensity [63]. CDR1 and CDR2 have a constant length of seven amino acids, while four different lengths of CDR3 (9, 12, 15 and 18 amino acids) were used with randomized sequence (except cysteine) at each position, mimicking natural sequence and length diversity. The library was generated by PCR assembly using primers with partially randomized sequence for the CDR regions following standard protocols.

#### Ribosome display in vitro selection

Ribosome display using this synthetic nanobody library then was performed against ADDoCoV (comprising the SARS-CoV-2 S RBM AH epitope) as described [26]. After five cycles of ribosome display against ADDoCoV immobilized on 96-well microtiter plates, the DNA pool was cloned into pHEN6. Individual colonies were picked, and nanobodies expressed in *E. coli* TG1 (Agilent Technologies) in dYT medium at 30°C overnight after induction with 1 mM IPTG. Nanobodies binding ADDoCoV, the SARS-CoV-2 RBD and S, but not ADDomer alone or a mutant S devoid of AH (SpikeΔAH), were identified by ELISA and then sequenced (Table S3).

### SPR experiments

#### ADAH11 nanobody binding to SARS-CoV-2 RBDs

Interaction experiments using SPR of ADAH11 nanobody monomer and different RBDs were carried out with a Biacore T200 system (GE Healthcare) according to the manufacturer’s protocols and recommendations. Briefly, biotinylated RBD proteins were immobilized on streptavidin-coated SA sensor chips at ~3845 response units (RU) for Ancestral RBD and ~2500 RU for Delta and Omicron RBD. Binders were diluted to the concentrations indicated (Fig. 2G) and passed over immobilized RBDs at a flow rate of 30 μL/min. The Running Buffer for all SPR measurements was PBS. The sensorgrams were analyzed using the Biacore Evaluation Software (GE Healthcare) and *k*_on_, *k*_off_ and K_D_ values were determined using a two-state reaction binding model. All experiments were performed in triplicates.

#### Gigabody binding to Ancestral S RBD

Purified, biotinylated Ancestral S RBD ligand was immobilized on a streptavidin-coated (SA) sensor chip (GE Healthcare) at 2453 RU. For all interaction measurements, the analyte was injected at a flow rate of 50 μL/min for 120 s using PBS as the Running Buffer. Dissociation was performed for 600 s. Gigabody, and AdY25 ADDomer as a negative control, were serially diluted and injected at concentrations of 0.5, 1.0, 1.5 and 2.0 nM. The chip was regenerated using 2 injections of 10 mM glycine pH 2.6. All measurements were performed in triplicates. Final sensorgrams were obtained by subtracting the control sensorgrams from the corresponding Gigabody sensorgrams accounting for nonspecific binding to the sensor chip. Fitting with Biacore Evaluation Software (GE Healthcare) indicated picomolar binding (K_D_ = 30 ± 20pM) dictated by very slow dissociation kinetics.

#### Gigabody binding to Omicron S RBD

Purified, biotinylated Omicron RBD ligand was immobilized on a SA sensor chip (GE Healthcare) at 3622 RUs. Injection, dissociation and regeneration were performed as above, for Gigabody serial diluted at concentrations of 1.0, 1.5, 2.0 and 2.5 nM. Sensorgrams were analyzed with the Biacore Evaluation Software, again indicating picomolar binding (K_D_ = 10 ± 3pM) with very slow dissociation kinetics, similar to Ancestral S RBD.

### Molecular dynamics simulations

#### Construction of a complete ADDoCoV model using Rosetta and MD

The input model was based on the cryo-EM structure combined with the AH-epitope sequence (Fig. 1D, Table S1) added manually adopting a structure derived from the ACE2 receptor in complex with the RBD of the S protein (PDB ID 7C8D) [64]. The RGD loop, unresolved in the cryo-EM density, was reconstructed using Rosetta [65–69]. Symmetrical pentamer models were generated with Rosetta SymDock [67, 68], and the introduced 5-fold symmetry was maintained during all following steps. Missing loops were reconstructed using Rosetta Remodel [65, 69]. Models were relaxed using Rosetta Relax and subjected to MD simulations with GROMACS 2019 [70]. The ADDoCoV structure was parametrized with the gromos54a7 forcefield in a cubic box with simple point charge water and sodium ions to neutralize the net charge. MD comprised five replicates of 100 ps of NVT followed by 67 ns of NPT simulations. Trajectories were analyzed with CPPTRAJ [71]. All analyses were based on Cα positions if not stated otherwise. The first 10 ns of each production MD run were excluded from all analyses to allow time for system equilibration. The conformational landscape of the AH epitope was analyzed by principal component analysis (MDTraj [72] and sklearn [73]) and cluster analyses (CPPTRAJ, kmeans algorithm) based on the same cartesian space.

#### Construction of a Gigabody model using Rosetta and MD

Missing loops in the ADDomer model, sequence adjusted for the AdY25 penton base protomer (Table S1), were constructed using Rosetta SymDock [67, 68], Remodel [65, 69] and Relax [66] as described for the ADDoCoV model. The trimeric ADAH11 nanobody fiber tag structure was modeled based on the bacteriophage T4 fibritin derived trimeric foldon structure (PDBID: 4NCV) [74]. The structures of the fiber tail peptide fused to the N-terminus of the foldon, and ADAH11 nanobody fused to the C-terminus, were predicted with trRosetta [75]. Two amino acids of the foldon were included during the prediction with trRosetta at the C-terminus of the fiber tail peptide and the N-terminus of the nanobody, respectively (Fig. S11). The fiber tail peptides and nanobodies were aligned to the trimeric foldon using these overlapping residues. The complete ADAH11-Trimer structure was subsequently relaxed with Amber [71] using the ff14SB forcefield and no solvent in three consecutive minimizations with 1000 cycles with positional restraints on all atoms of 10, 1 and 0 kcal/mol/A^2^.

Next, the structure was further relaxed with Rosetta [66] using the MonomerRelax2019 script and the 10 lowest energy structures were placed manually in PyMol on top of the AdY25 ADDomer A57S model guided by the Ad5 penton base fiber tail peptide complex (PDB ID 1X9T) [30]. Subsequently, Amber [71] was used to relax the fiber tail peptide to the position observed in the experimental structure (PDB ID 1X9T) [30]. A total of 50 ns MD simulations were then performed with Amber. The protein was parametrized with the united-atom forcefield ff03u [76]. The ADDomer-fiber tail peptide complex was relaxed with increasing positional restraints (1000 steps, ntmin = 3, restraint_wt = 0.1, 0.2, 0.5, 1, 2, 5 and 10) on the ADDomer and the fiber tail peptides to generate a complex in the experimentally observed conformation (PDB ID 1X9T) [30].

Two additional minimization steps were performed, the first without positional restraints and the second with implicit Born solvation model (IGB = 1) [77]. After an initial heating step (0.05 ns from 0.1 to 300 K), 50 ns MD simulations were performed with 2 fps timestep using implicit solvation and Langevin dynamics (ntt = 3) for each of the 20 starting structures (10 Rosetta models ^*^ 2 conformations). Four runs with the three fiber tail peptides in proximity and three runs with two peptides in proximity were unstable and discarded. To illustrate the full scale of the Gigabody (Fig. 3C, Fig. S11), the modeled pentamers from these simulations were aligned with the pentamers in the ADDomer cryo-EM structure (PDB ID 6HCR) [23].

### SARS-CoV-2 MiniV preparation

Artificial minimal SARS-CoV-2 virions (MiniVs) were assembled from small unilamellar vesicles (SUVs) as described previously [33]. Briefly, SUVs containing NTA(Ni^2+^) and Rhodamine B-functionalized membranes were coupled to recombinant Ancestral SARS-CoV2 S ectodomains bearing an oligohistidine tag [29]. SUVs were prepared by membrane extrusion to obtain a monodisperse vesicle population with a mean diameter of 100 nm from a lipid solution of 45 mol% DOPC, 21 mol% DOPE, 3 mol% DOPS, 12 mol% DOPI, 14 mol% cholesterol, 3 mol% SM, 1 mol% DGS-NTA(Ni^2+^) and 1 mol% Rhodamine B-PE (all lipids obtained from Avanti Polar Lipids).

MiniV size distribution was measured by dynamic light scattering with a Malvern Zetasizer Nano ZS system at a total lipid concentration of 100 μM in PBS. Temperature equilibration time was set to 300 s at 25°C, followed by three repeated measurements for each sample at a scattering angle of 173° using the built-in automatic run-number selection. The material refractive index was set to 1.4233 and solvent properties to *η* = 0.8882, *n* = 1.33 and *ε* = 79.0. For assessment of Gigabody-mediated MiniV clustering, the MiniV solution was preincubated with 1.5 nM Gigabody for 30 min in the dark at 4°C before measurement.

For confocal microscopy observation of MiniV-cell attachment after 2.5 h of incubation under control conditions, or with addition of 500 nM nanobodies or 1.6 nM Gigabody, respectively, A549 cells stably expressing ACE2 [78] were stained with CellTracker Green CMFDA dye (Invitrogen, USA) according to the manufacturer’s recommendations. Nuclei were stained with 10 μM Hoechst33342 (Sigma Aldrich). Laser scanning confocal microscopy was performed with a LSM 800 (Carl Zeiss AG). Images were acquired with a ×63 immersion oil objective (Plan-Apochromat ×63/1.40 Oil DIC, Carl Zeiss AG). Analysis was performed with ImageJ (NIH) and adjustments to image brightness and contrast, as well as background corrections, were always performed on the whole image and special care was taken not to obscure or eliminate any information from the original image.

### SARS-CoV-2 MiniV retention assays

Retention assays were performed as described previously [33] using human ACE2 expressing A549 cells. Briefly, MiniVs were incubated with A549 cells at a final lipid concentration of 10 μM in flat bottom 96 well plates and in low serum containing culture medium (DMEM supplemented without phenol red, 4.5 g/l glucose, 1% L-glutamine, 1% penicillin/streptomycin, 0.01 mg/mL recombinant human insulin and 0.5% fetal bovine serum). After 2.5 h, MiniV Rhodamine B fluorescence was measured with a plate reader for each well in four positions. Cultures were afterwards washed 3 times with PBS. Subsequently, residual fluorescence was measured in each well and normalized to the initial fluorescence intensity to calculate MiniV retention values after correction for background fluorescence and negative controls. Gigabody dilution curves for retention analysis were prepared by preincubating MiniVs with 1.6 nM Gigabody for 30 min at 4°C in the dark before addition to the cells.

### Mouse immunization experiments

Female C57BL/6 mice were obtained from Charles River Laboratories (UK) and maintained at the University of Bristol Animal Services Unit in specific pathogen-free conditions in accordance with established practices and under a UK Home Office License [79]. Mice were immunized with 40 μg ADDoCoV vaccine or ADDomer scaffold as a control via IN, IM or SC routes (*n* = 10 mice per treatment group pooled across two experimental replicates) on Day 0 (primary immunization), Day 21 (boost 1) and Day 42 (boost 2). Mice were humanely euthanized on Day 62; 9 weeks post initial immunization, by terminal exsanguination under general anesthesia.

#### Intranasal

Mice were lightly anaesthetized using isoflurane, and 12.5 μL ADDoCoV vaccine in sterile PBS (1.6 mg/mL) was instilled into each nostril (total dose 25 μL; 40 μg).

#### Intramuscular

Mice were lightly anaesthetized using isoflurane and received IM injection with 50 μL ADDoCoV vaccine in sterile PBS (0.8 mg/mL) into the quadriceps muscle using a 25G 5/8-inch needle (total dose 50 μL; 40 μg).

#### Subcutaneous

Non-anaesthetized mice were restrained in a tube restrainer. SC injection was performed with 50 μL ADDoCoV vaccine in sterile PBS (0.8 mg/mL) using a 25G 5/8-inch needle at the tail base (total dose 50 μL; 40 μg).

#### Sample collection

The presence of serum antibody was assayed in peripheral blood at baseline (Day -1), Day 20, and Day 41 and in terminal bleeds on Day 62. Peripheral blood samples (30–50 μL) were collected from the lateral tail vein. For collection of terminal blood samples, mice were deeply anesthetized using isoflurane, and 500–800 μL of blood was collected following thoracotomy and cardiac puncture. Peripheral blood and terminal bleed samples were processed for serum collection. Blood was collected into autoclaved microcentrifuge tubes without anti-coagulant and allowed to clot at room temperature for 20 min. Samples were centrifuged at 2000 g for 10 min at 4°C. Serum was transferred to a fresh microcentrifuge tube, and centrifugation was repeated at 2000 g for 10 min at 4°C. Following centrifugation, serum was transferred to a fresh microcentrifuge tube and frozen in aliquots at −80°C. NW and BAL were taken post-mortem using established methodology [80, 81].

The presence of mucosal antibody in murine nasal secretions was assayed by flushing a 500 μL volume of ice-cold PBS through the nasal turbinates. Briefly, scissors were used to make an incision from the abdomen to the jaw in order to expose the thoracic cage and neck. The trachea was exposed and a 20G × 32 mm Surflo intravenous catheter (VWR international) inserted. A 1 mL syringe containing 500 μL PBS was then attached and the fluid used to flush the nasal cavity. Fluid existing the nares was captured using an Eppendorf and then incubated on ice with Protease inhibitor cocktail (Roche Diagnostics). Washes were centrifuged at 1000 g for 10 min at 4°C to remove cellular debris and mucus [82]. Fluid supernatants were transferred to fresh autoclaved microcentrifuge tubes and immediately frozen in aliquots at −80°C.

To isolate mucosal antibody in the lower respiratory tract, lung lavages were performed. Briefly, a 20G intravenous catheter with stylet withdrawn was inserted, directed toward the lungs. A syringe containing 1 mL ice-cold PBS was used to aspirate the lungs, ensuring not to overinflate and rupture the tissue. To prevent PBS from leaking from the catheter insertion site, thread was used to tie off the catheter to the trachea. Recovered PBS from lung washes were again incubated on ice with protease inhibitor then centrifuged at 1000 g for 10 min at 4°C to remove cellular debris and mucus [81]. Fluid supernatants were transferred to fresh autoclaved microcentrifuge tubes and immediately frozen in aliquots at −80°C.

### Enzyme-linked immunosorbent assay

#### Antigen-specific serum antibody ELISA

The antigen-specific IgG and IgA titres in mouse sera were assessed by a semi-quantitative ELISA. MaxiSorp high binding ELISA plates (Nunc) were coated with 100 μL/well of 1 μg/mL highly purified SARS-CoV-2 RBDs. For the IgG and IgA standards, plates were coated with 1:1000 dilution each of goat anti-mouse Kappa (Catalog #1050-01, Southern Biotech) and Lambda light chains (Catalog #1060-01, Southern Biotech). After overnight incubation at 4°C, the plates were washed 4 times with PBS-Tween 20 0.05% (v/v) and blocked for 1 h at 37°C with 200 μL/well blocking buffer (1% BSA (w/v) in PBS-Tween-20 0.05%(v/v)). The plates were then washed, and 10-fold serial dilutions of serum samples (10^3^–10^6^), or a 5-fold dilution series starting at 200 ng/mL of purified IgG (Catalog #0107-01, Southern Biotech) or IgA (Catalog #0106-01, Southern Biotech) were added using 50 μL/well volume. Plates were incubated for 1 h at 37°C, then washed and secondary antibody added at 1:2000 or 1:4000 dilution in blocking buffer (100 μL/well) using either anti-mouse IgG-HRP (Catalog #1030-05, Southern Biotech), or anti-mouse IgA-biotin (Catalog #1040-80, Southern Biotech). After a 1 h incubation at 37°C, plates incubated with biotinylated antibody were washed and incubated at 37°C for 1 h with a 1/200 dilution of Streptavidin-HRP (Catalog #890803, R&D systems). Plates were then washed and developed using 100 μL/well SureBlue TMB (3,3′, 5,5′-tetramethylbenzidine) substrate, and the reaction stopped after 5 min with 100 μL/well stop solution (Insight Biotechnologies). The absorbance was read on a FLUOstar Omega multi-mode microplate reader at 450 nm (BMG LABTECH). For assaying mucosal samples for the presence of antigen-specific IgG and IgA antibody, the same procedure was followed except mucosal samples were used at a 1/10–1/250 dilution series. To determine the presence of cross-reactive antibody in the serum or mucosal secretions of vaccinated mice, the binding of antibody to different variants of SARS-CoV-2 RBD (Ancestral, Alpha, Beta, Delta and Omicron) was measured. Here, the variant RBDs were used to coat MaxiSorp high binding plates and ELISA performed as before.

#### Nanobody ELISA

Proteins ADDomer, ADDoCoV, RBD, S, SpikeΔAH and BSA were produced and purified as described [52]. Highly purified proteins were diluted in PBS to a final concentration of 40 μg/mL. Next, 100 μL of the diluted proteins were added to the corresponding well in a microtiter plate followed by a gentle tap to ensure even coating of all wells before sealing the plate and incubating overnight at 4°C. On the following day, supernatants were discarded, and the plate washed 3 times with 300 μL Wash Buffer (PBS pH 7.4, 0.1% Tween) before drying the plate by placing it upside down on a paper towel to remove residual Wash Buffer. Next, 200 μL Blocking Solution (PBS pH 7.4, 5% milk) was added to each well, and the plate was incubated at room temperature for 1 h. The nanobody samples were diluted in Wash Buffer to a concentration of 1 μM concentration. Subsequently, the Blocking Buffer was removed from plate before drying on a paper towel, followed by adding 100 μL of corresponding nanobodies or control buffer, respectively, to each well of the plate before incubating the plates at room temperature for 1 h. Next, the samples were removed from the plate, and the plate was washed with 300 μL Wash Buffer 3 times and then dried on paper towel. Finally, 50 μL of anti FLAG-HRP antibody (dilution 1:3000) was added to each well, and the plate was incubated at room temperature for 1 h. Sample was removed, and the plate was washed with 300 μL Wash Buffer 3 times and dried on a paper towel. Then, 100 μL of TMB reagent was added to each well, followed by incubation at room temperature for 5 min and stopping the reaction with addition of 50 μL of 1 N HCl to each well. Finally, absorbance at 450 nm was measured in a microplate reader. The data were plotted using Microsoft excel. The standard deviation of triplicates was added as error bars.

### SARS-CoV-2 virus neutralization assay

Vero E6 cells engineered to express the cell surface protease TMPRSS2 (VeroE6-TMPRSS2) [83] (NIBSC) and Caco-2 cells engineered to express ACE2 [84] were cultured at 37°C in 5% CO_2_ in DMEM containing GlutaMAX (Gibco, Thermo Fisher) supplemented with 10% (v/v) FBS (Gibco) and 0.1 mM non-essential amino acids (NEAA, Sigma Aldrich). The ADAH11 nanobody was serially diluted 2-fold for eight dilutions, from a 0.85 μg/mL starting dilution, in triplicate, in Minimum Essential Media (MEM, Gibco) containing 2% (v/v) FBS and NEAA. The ancestral SARS-CoV-2 isolate hCoV-19/England/02/2020 (GISAID ID: EPI_ISL_407073) was grown on VeroE6-TMPRSS2 cells and titrated as previously described [80]. Virus (60 μL of 8 × 104 TCID50/mL) was mixed 1:1 with dilutions of ADAH11 and incubated for 60 min at 37°C. Following the incubation, supernatants were removed from Caco-2-ACE2 and VeroE6-TMPRSS2 cells seeded previously in μClear 96 well microplates (Greiner Bio-One) and replaced with 100 μL of the virus:sera dilutions followed by incubation for 18 h at 37°C in 5% CO_2_. Control wells containing virus only (no ADAH11) as well as a positive control (a commercial monoclonal antibody (Absolute Antibody; Sb#15) recognizing the S protein RBD) and media only negative control were also included on each plate. Cells were fixed by incubation in 4% paraformaldehyde for 60 min followed by permeabilization with Triton-X100 and blocking with BSA. Cells were stained with DAPI (Sigma Aldridge) and an antibody against the SARS-CoV-2 nucleocapsid protein (1:2000 dilution, 200-401-A50, Rockland) in combination with a corresponding fluorophore conjugated secondary antibody (Goat anti-Rabbit, AlexaFluor 568, Thermo Fisher). Images were acquired on the ImageXpress Pico Automated Cell Imaging System (Molecular Devices) using a 10X objective. Stitched images of nine fields covering the central 50% of the well were analyzed for infected cells using Cell ReporterXpress software (Molecular Devices). Cell numbers were determined by automated counting of DAPI stained nuclei, infected cells were determined as those cells in which positive nucleocapsid staining, associated with a nucleus, was detected. The percentage of infected cells relative to control wells containing virus only (no ADAH11) were calculated.

### Surrogate virus neutralization assay

Remaining samples of sera from corresponding administration routes along with prebleed samples were pooled and passed through a protein A column and the recovered IgGs used in SARS-CoV-2 sVNT [35] using a commercial kit (GenScript).

**Statistics**. Statistical significance was determined by calculating standard deviations following standard mathematical formulae**.** For biochemical experiments, standard deviations were calculated from independent triplicates unless indicated otherwise. For mouse immunization data, statistical analyses were carried out using a Mann–Whitney nonparametric *t* test and GraphPad Prism software.

## Supplementary Material

Supplement_ADDoCoVGigabody_Revised_01092023_PDF_tbad024

Movie_S1_ADDoCoV_Nanoparticle_Vaccine_reduced_tbad024

Movie_S2_Gigabody_Nanoparticle_Superbinder_reduced_tbad024

## Data Availability

All data needed to evaluate the conclusions in the paper are present in the paper and/or the Supplementary Materials. All datasets generated during the current study have been deposited in the Electron Microscopy Data Bank (EMDB) under accession numbers EMD-16512 (ADDoCoV), EMD-16522 (ADDoCoV-ADAH11), and in the Protein Data Bank (PDB) under accession number PBD ID 8C9N (ADDoCoV). Reagents are available from F.G., C.S. and I.B.

## ANIMAL RESEARCH STATEMENT

Animal studies were conducted at the University of Bristol in facilities licensed under the Animal Scientific procedures Act (PEL XA320B4BF), covered by a license from the British Home Office (PPL PP1105873), and complying with EU Directive 2010/63/EU. Procedures were carried out according to the principles in the Guide for the Care and Use of Laboratory Animals (The Institute of Laboratory Animal Resources, 1996). Termination was conducted according to humane methods outlined in the Guidance on the Operation of the Animals (Scientific Procedures) Act 1986 Home Office (2014).
