# Supplementary material for: In vitro generated antibodies guide thermostable ADDomer nanoparticle design for nasal vaccination and passive immunization against SARS-CoV-2
Source: Antib Ther. 2023 Oct 17;6(4):277–97. doi: 10.1093/abt/tbad024 (PMC10702856; doi:10.1093/abt/tbad024)
Supplement: Supplement_ADDoCoVGigabody_Revised_01092023_PDF_tbad024 [file supplement_addocovgigabody_revised_01092023_pdf_tbad024.pdf]

Supplementary Materials for

In vitro generated antibodies guide thermostable ADDomer nanoparticle design for nasal vaccination and passive immunization against SARS-CoV-2

Dora Buzas[1,2], H. Adrian Bunzel[2], Oskar Staufer[1,3], Emily J. Milodowski[4], Grace L. Edmunds[4], Joshua C. Bufton[2], Beatriz V. Vidana Mateo[4], Sathish K. N. Yadav[2], Kapil Gupta[2,6], Charlotte Fletcher[2], Maia Kavanagh Williamson[5], Alexandra Harrison[2], Ufuk Borucu[2], Julien Capin[2], Ore Francis[4], Georgia Balchin[2], Sophie Hall[2], Mirella Vivoli Vega[2], Fabien Durbesson[7], Srikanth Lingappa[2], Renaud Vincentelli[7], Joe Roe[4], Linda Wooldridge[4], Rachel Burt[4], J. L. Ross Anderson[2], Adrian J. Mulholland[8], Bristol UNCOVER Group[9], Jonathan Hare[6], Mick Bailey[4], Andrew D. Davidson[5], Adam Finn[9,10], David Morgan[5], Jamie Mann[4], Joachim Spatz[1,11], Frederic Garzoni[6*], Christiane Schaffitzel[2,9*], Imre Berger[1,2,8,9*]

[*]Correspondence to: fred@imophoron.com, cb14941@bristol.ac.uk, imre.berger@bristol.ac.uk

This Supplement PDF file includes:

Figs. S1 to S11

Tables S1 to S6

Captions to Movies S1, S2

Other Supplementary Material for this manuscript includes the following:

Movies S1, S2

Fig. S1

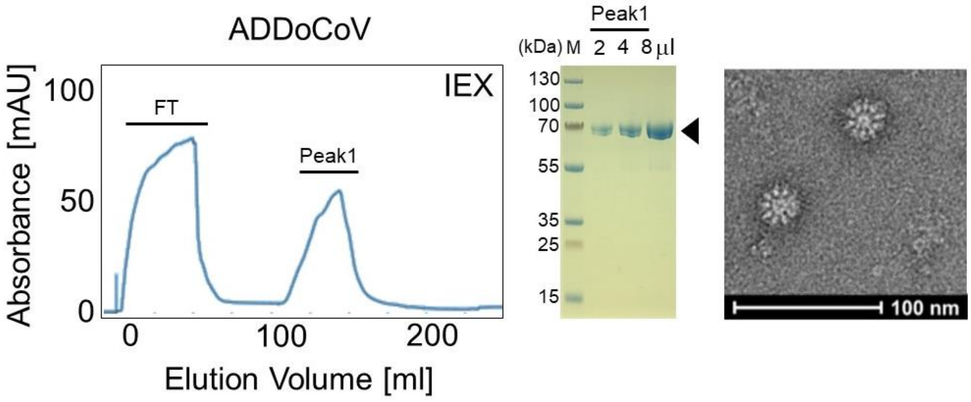

Purification and quality control of ADDoCoV. Ion exchange (IEX) chromatogram of ADDoCoV S protein using a Bio-Scale Mini Macro Prep High Q column (left). Absorption was detected at 280 nm (blue line). Flow through (FT) and peak fractions are indicated. ADDoCoV protein (Peak 1) was confirmed by the SDS-PAGE analysis (middle) of pooled Peak 1 fractions. Aliquot volumes loaded are indicated. M stands for molecular weight marker, molecular weights (in kDa) corresponding to marker bands are indicated. Negative-stain EM micrograph of Peak 1 is shown (right, scale bar: 100 nm). This material was used for negative-stain EM and cryo-EM sample preparations.

Fig. S2

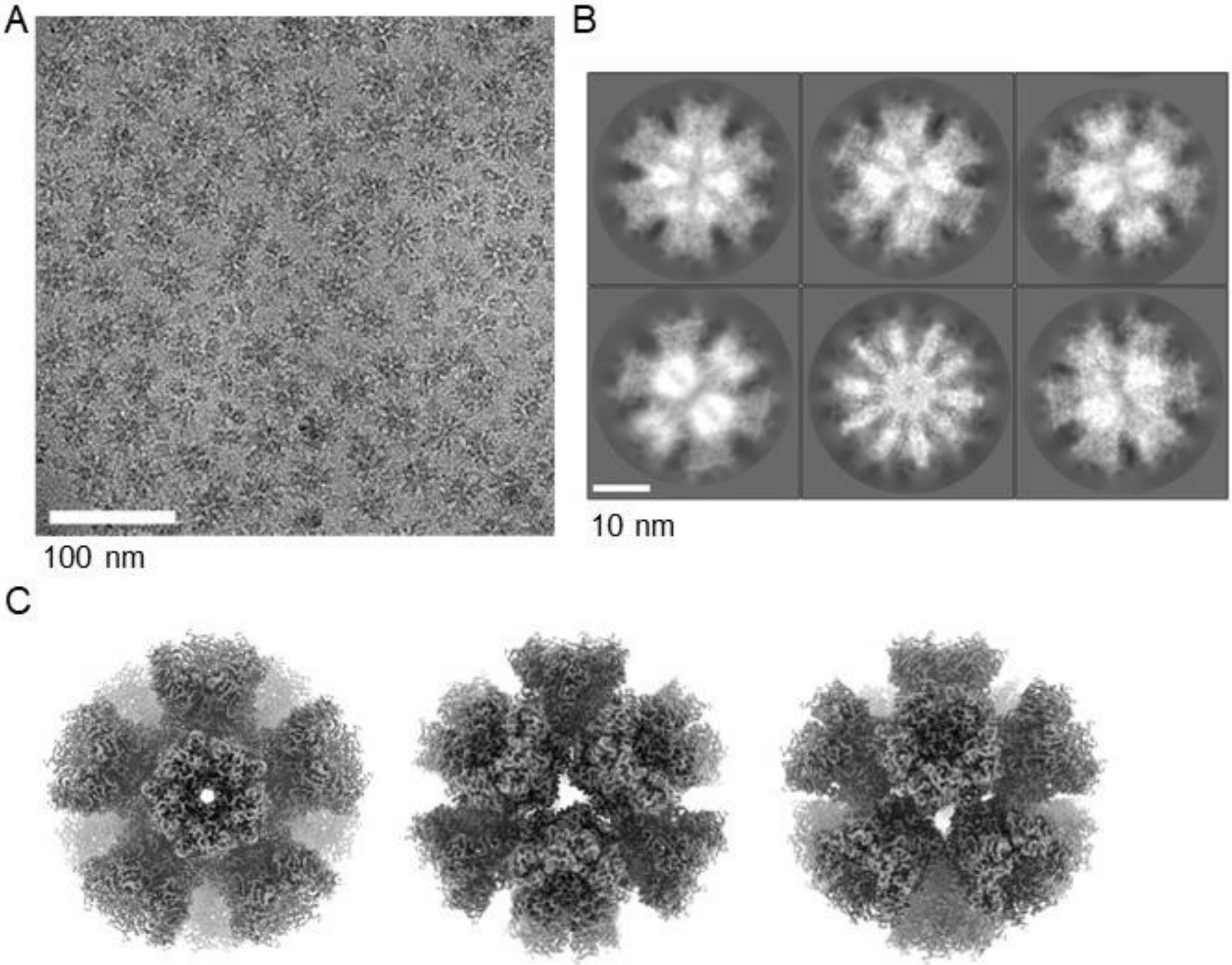

Electron microscopy of ADDoCoV. **(A)** A representative cryo-EM micrograph with ADDoCoV particles is shown. Some dissociation of ADDoCoV particles into pentons is observed during cryo-grid preparation. **(B)** Reference-free 2D class averages from RELION 3.1. Scale bars are indicated. **(C)** Final cryo-EM map with icosahedral symmetry at 2.36 Å. Three different views are shown.

Fig. S3

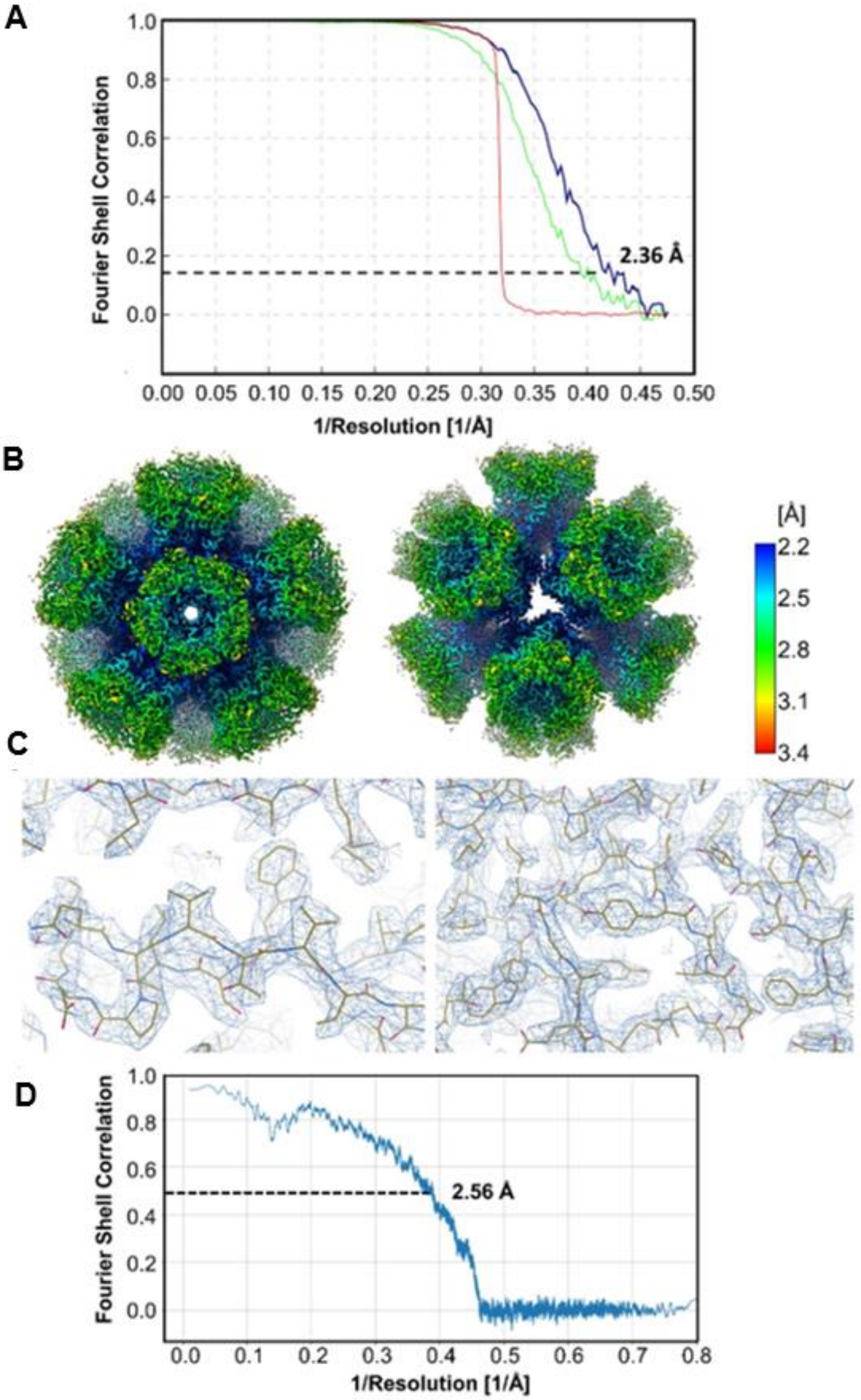

Quality of the ADDoCoV map and model. (A) The Fourier Shell correlation (FSC) curve after gold-standard refinement of 32,227 particles (blue curve). The FSC = 0.143 criterion indicates an overall resolution of 2.36 Å. Green curve: FSC curve of unmasked maps; red curve: FSC curve of phase

randomized masked maps. **(B)** Local resolution of the final ADDoCoV cryo-EM map calculated in RELION 3.1. The core of the complex is resolved at 2.2 Å whereas peripheral parts comprising the VL and RGD loops have a lower resolution of ~ 3-3.2 Å. **(C)** Representative EM density of the ADDoCoV containing the refined atomic model. **(D)** FSC curve calculated between the atomic model and the final cryo-EM map. The map/model FSC at 0.5 reaches a resolution of 2.56 Å.

Fig. S4

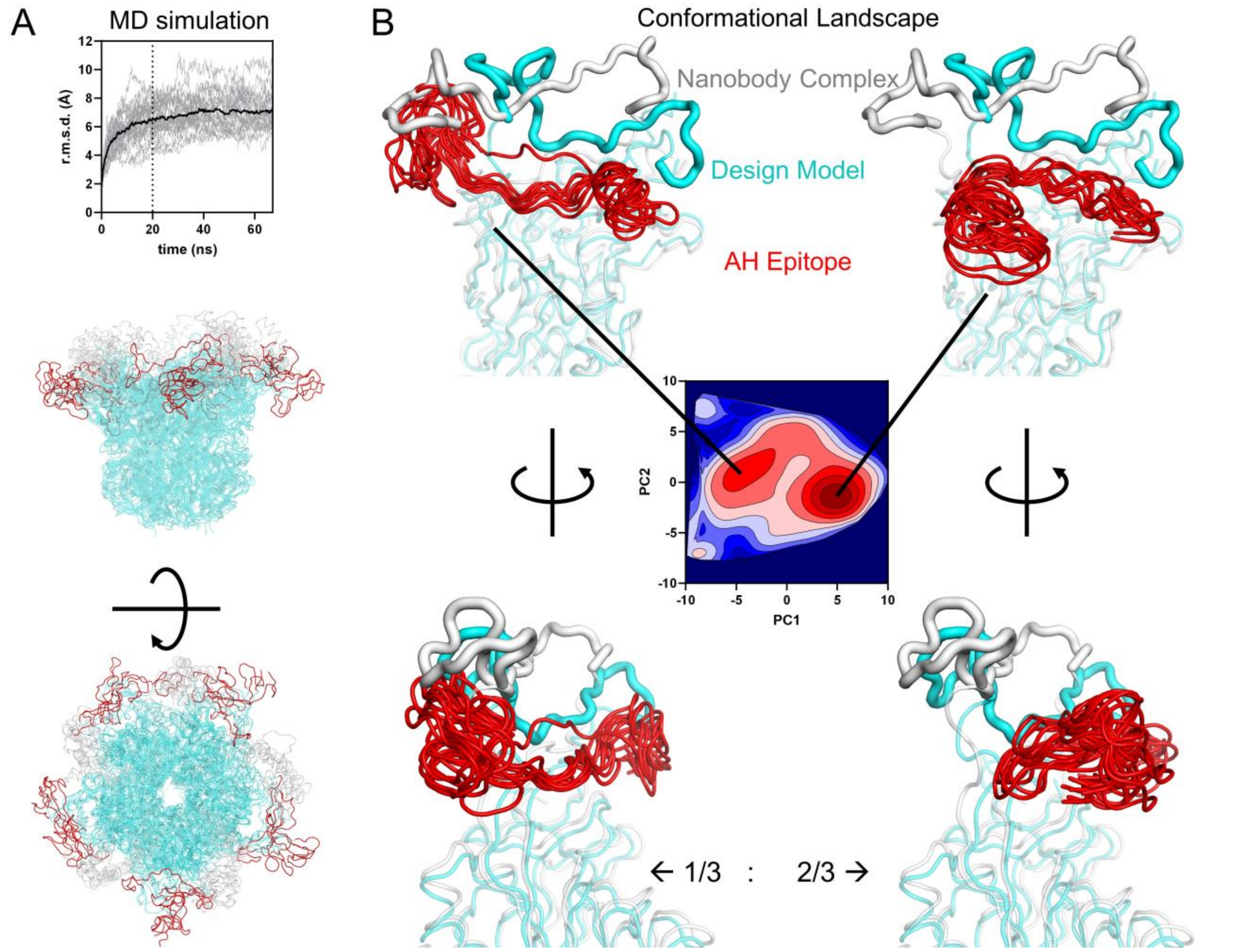

Dynamics of ADDoCoV. (**A**) MD simulations of the ADDoCoV protomer were performed based on the Rosetta Model (AH-epitope: red; RGD-loop: grey; Scaffold: cyan). As expected, the AH-epitope and RGD-loop were flexible, whereas the overall protein (cyan) remained similarly structured to its cryo-EM model. (**B**) The dynamics of the AH-epitope were analyzed for each monomer separately, resulting in an accumulated simulation time of 5 monomers x 5 replicates x 65 ns, which allowed for analysis of the conformational dynamics of the epitope in detail by principal component analysis and cluster analysis. The AH-epitope explores a broad and shallow conformational landscape with two pronounced minima. 1/3 of the trajectory populated a minimum corresponding to a conformation similar to the designed model (cyan) resembling the conformation in the open SARS-CoV-2 S form. The other 2/3 of the population corresponds to a flipped-down conformation, in which a part of the epitope protrudes sideways into the space in between pentons.

Fig. S5

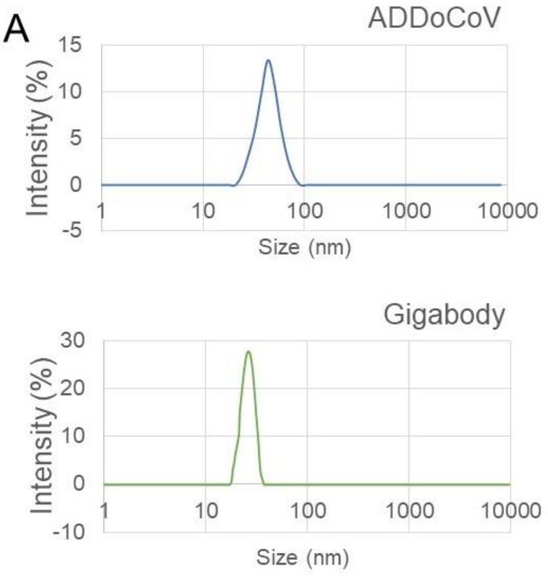

Nanoparticle biophysics. (A) Size distribution of ADDoCoV nanoparticle vaccine (colored in blue) and Gigabody nanoparticle (colored in green) are shown. The polydispersity index (PDI) is 0.16 for ADDoCoV and 0.14 for Gigabody, respectively. **(B)** Zeta potentials of ADDoCoV (blue) and Gigabody (green) are shown in a bar diagram. Standard deviations are indicated by error bars.

Fig. S6:

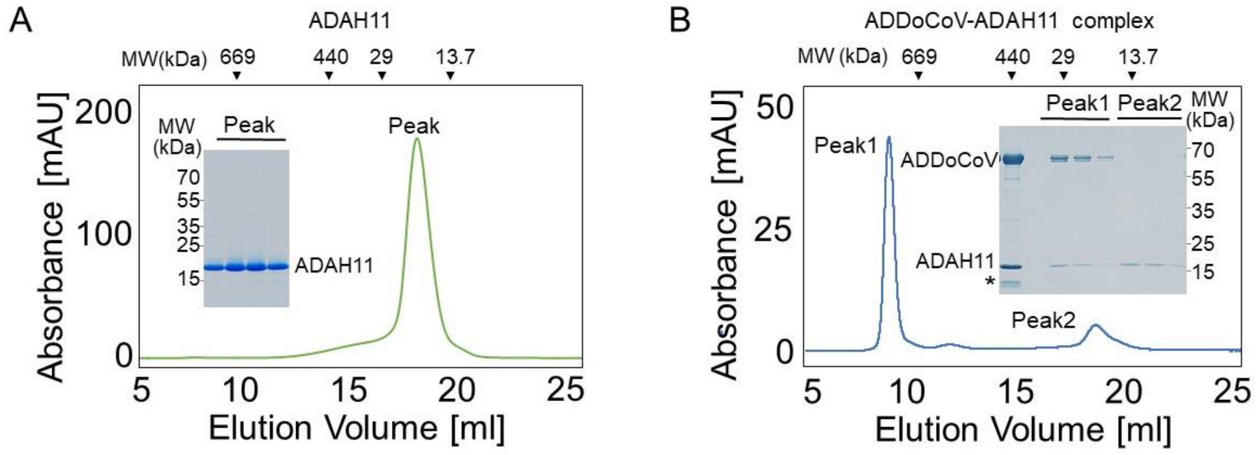

ADAH11 nanobody binding to ADDoCoV. (A) Size exclusion chromatography of purified ADAH11 using a Superdex 200 column is shown. Inset: Coomassie stained SDS PAGE section showing the peak fractions. **(B)** Size exclusion chromatography of ADAH11 and ADDoCoV using a Superdex 200 column. Inset: SDS-PAGE gel showing the input mixture (lane 1), Peak 1 with bands corresponding to the complex of ADAH11 and ADDoCoV and Peak 2 corresponding to excess ADAH11. The star corresponds to a degradation product of the nanobody in the input mix.

Fig. S7

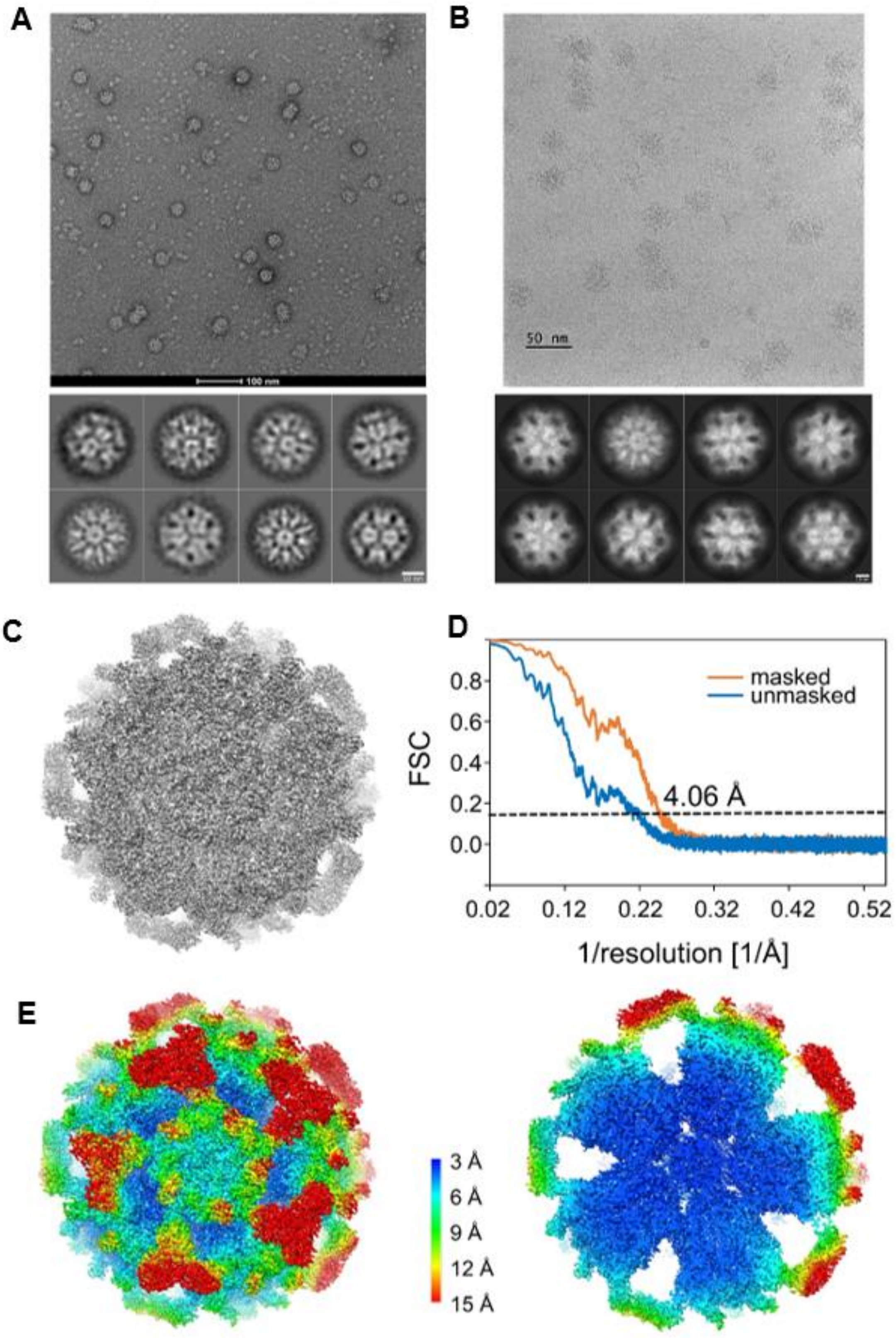

Electron microscopy of ADDoCoV-ADAH11 nanobody complex. (A) Negative stain micrograph showing purified ADDoCoV-ADAH11 complex and reference-free 2D class averages. Scale bars

correspond to 100 nm and 10 nm, respectively. **(B)** A representative cryo-EM micrograph with ADDoCoV-ADAH11 nanobody complex and reference-free 2D class averages. Scale bars correspond to 50 nm and 10 nm, respectively. **(C)** Final Cryo-EM map of the ADDoCoV-ADAH11 complex. No symmetry was applied. **(D)** The Fourier Shell correlation (FSC) curve after gold-standard **refinement**. The FSC = 0.143 criterion indicates an overall resolution of 4.06 Å. Blue curve: FSC curve of unmasked maps; orange curve: FSC curve of phase randomized masked maps. **(E)** Local resolution of the final ADDoCoV-ADAH11 complex cryo-EM map calculated in RELION 4.0. The core of the complex is resolved at ~3 Å whereas peripheral parts comprising the VL loop and the bound nanobody have a lower resolution of ~ 10-15 Å which is insufficient to build an atomic model.

Fig. S8:

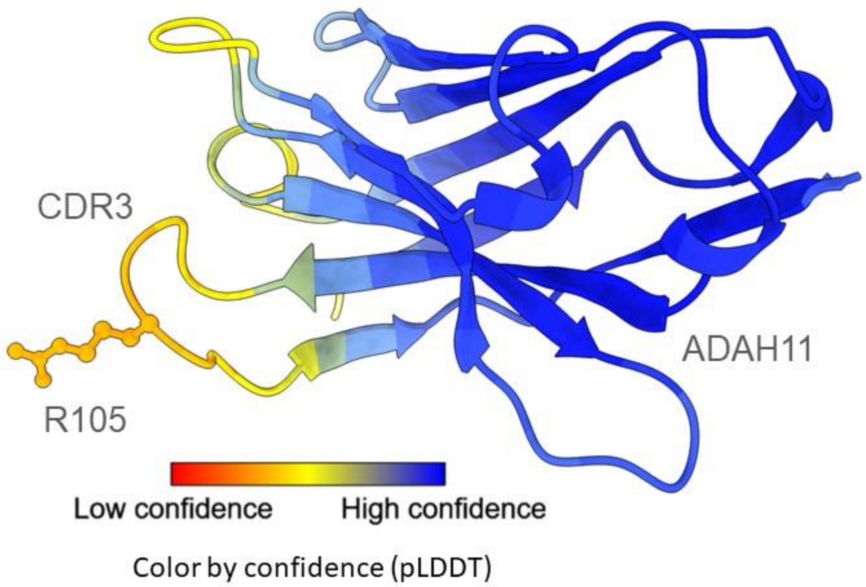

ADAH11 nanobody structural model. The structure of ADAH11 nanobody modelled using AlphaFold2 is shown in a ribbon representation, coloured according to the confidence of the prediction (blue, high confidence; red, low confidence) based on predicted local distance difference test (pLDDT) scores. The arginine residue at position 105 in the complementary determining region 3 (CDR3) is highlighted. The primary sequence of ADAH11 is provided in table S2.

Fig. S9

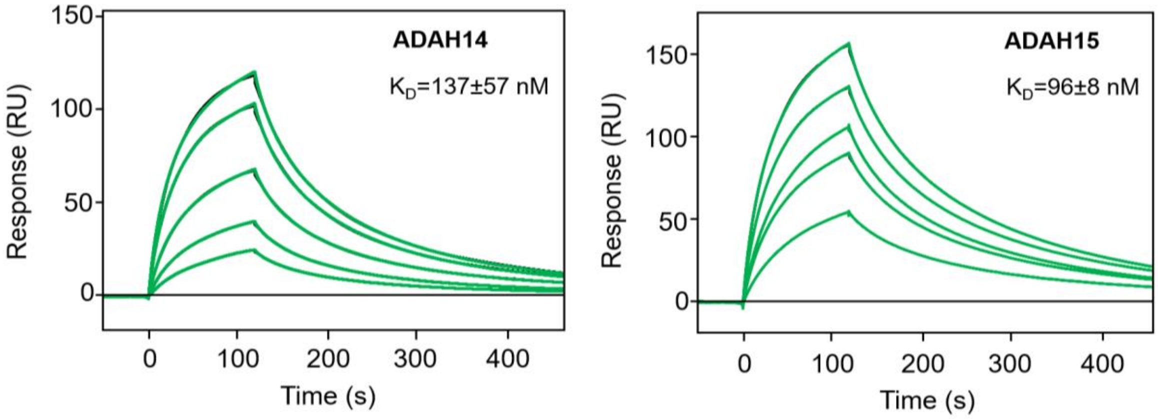

ADAH14 and ADAH15 binding to SARS-CoV-2 Ancestral RBD. Surface plasmon resonance of nanobodies ADAH14 (left) and ADAH15 (right) binding to immobilized Ancestral RBD. Nanobodies were injected in a concentration range from 50 nM to 250 nM. Data (green lines) were fitted (black lines) using the Biacore evaluation software.

Fig. S10

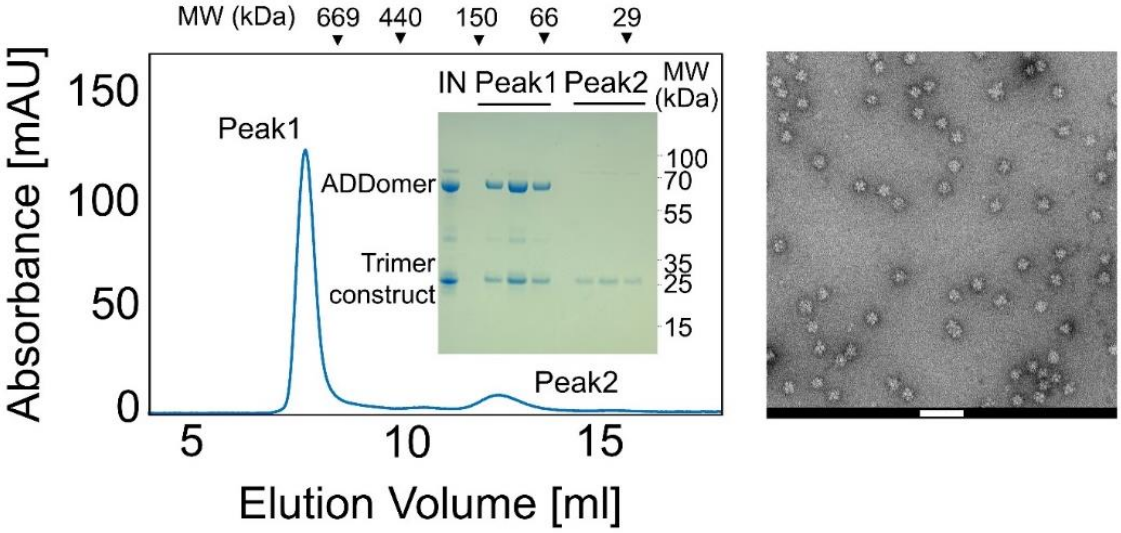

Purification and quality control of Gigabody. SEC of reconstituted Gigabody is shown (left). Molecular weight standard elution volumes are indicated (top). Gigabody elutes in Peak 1 close to the void volume. Coomassie-stained SDS gel showing input fraction, Peak 1 fractions with nanobody-trimer construct and penton base proteins and Peak 2 fractions containing nanobody only are shown in the inset. Negative stain EM of Peak 1 showing purified gigabodies is shown on the right. Scale bar corresponds to 100 nm.

Fig. S11

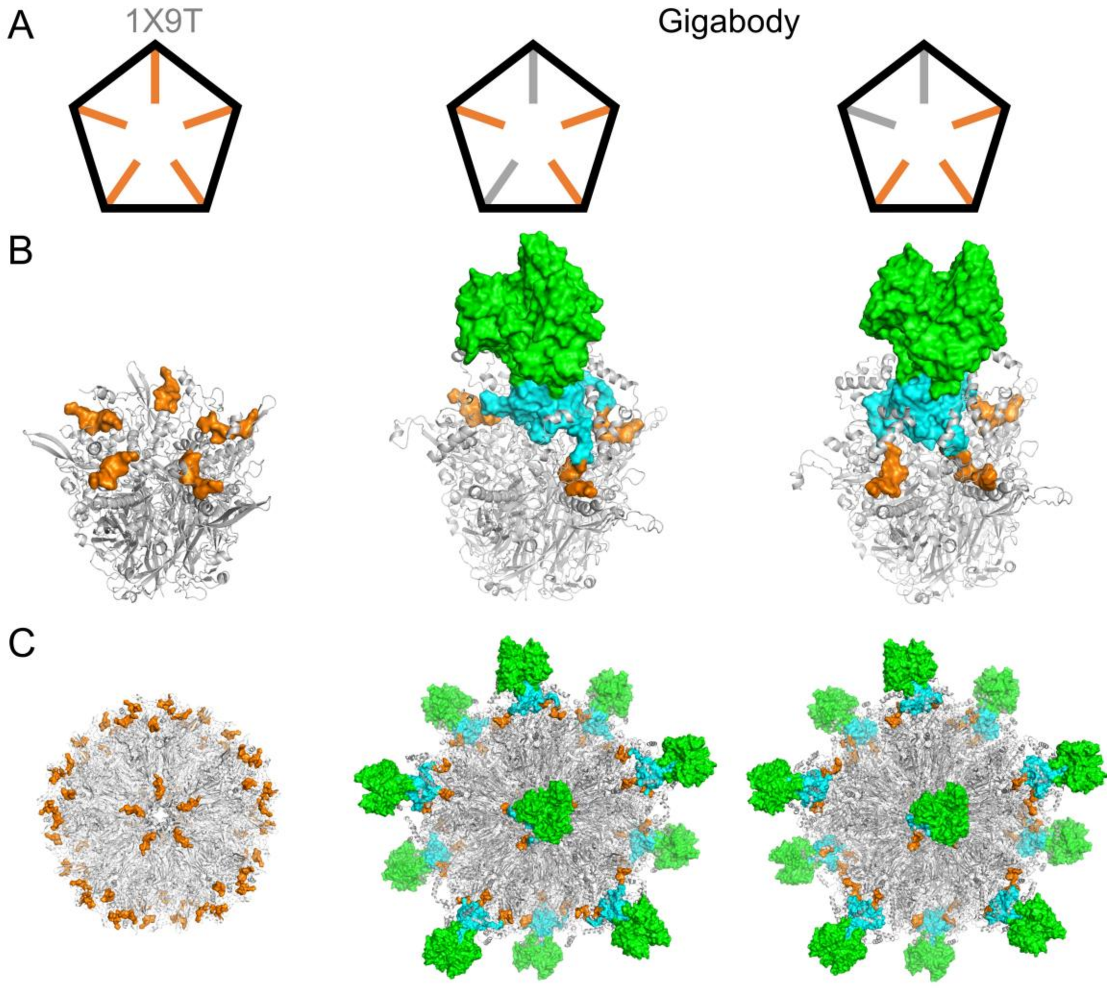

Gigabody modeling. Two distinct Gigabody models were constructed based on the Ad5 penton base fiber tail peptide complex structure (left, PDBID: 1X9T) with the fiber tails (orange) either maximally spaced (middle) or attached to adjacent penton base protomers on the penton (right). **(A)** Illustration of the observed binding modes of the fiber tail peptides (orange) on the pentons (circumference drawn in black). Instead of natural three fiber tail peptides of the trimeric fiber protein, five peptides are observed in the crystal structure occupying all possible binding sites. In Gigabody, due to ADAH11 trimer binding, two of the binding sites remain unoccupied (grey). **(B)** Model of the complex of the chimpanzee adenovirus derived ADDomer penton (grey) and the trimer formed by ADAH11 nanobody (green) linked to T4 foldon trimerization domain (blue) and fiber tail peptide (orange). **(C)** Model of the assembled 60mer, constructed by aligning the pentons to their position in the experimental Ad5 fiber tail complex structure.

Table S1:

Cryo-EM data collection and refinement statistics, ADDoCoV

**Data Collection and Processing**	
	ADDoCoV
Voltage (Kv)	200
Pixel size (Å/pix)	1.05 (0.525)
Nominal magnification	130,000
Exposure (e- /Å2)	44.03
Frames per exposure	40
Defocus range (µM)	-0.7 to -2.2
Final Particle Images	32,227
Symmetry imposed	I4
Resolution at FSC = 0.143 (Å)	2.36
Map-sharpening B factor ($Å^2$)	-25
**Atomic Model**	
**Model composition**	
Non-hydrogen atoms	211740
Protein residues	25980
**RMS deviations**	
Bond lengths (Å)	0.011
Angles	1.298
**Validation**	
MolProbity score	1.53
Clashscore	1.93
Rotamer outliers (%)	0
C-beta deviations	0
**Ramachandran plot**	
Favoured (%)	89.04
Allowed (%)	10.73
Outlier (%)	0.23

Table S2:

Sequences of nanobodies selected by Ribosome display.

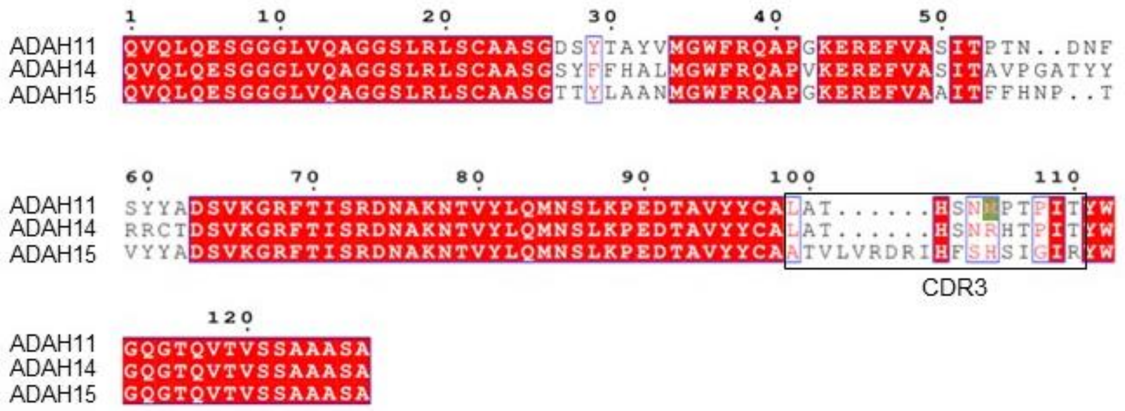

CDR3 boxed in black, ADAH11 R105 underlaid in green.

Supplementary Table S3:

Cryo-EM data collection statistics, ADDoCoV-ADAH11 complex

**Data Collection and processing**	
	ADDoCoV-ADAH11
Voltage (Kv)	200
Pixel size (Å/pix)	1.05 (0.525)
Nominal magnification	130,000
Exposure (e- /Å2)	55.6
Frames per exposure	45
Defocus range (µM)	-0.8 to -2.0
Final Particle Images	13950
Symmetry imposed	C1
Resolution at FSC = 0.143 (Å)	4.06
Map-sharpening B factor ($Å^2$)	-79.66

Table S4:

Adenoviral fiber tail peptide sequences

type	AA	Sequence	GenBank No.
AdY25	24	F N P V Y P Y	12657128
Ad12	18	F N P V Y P F	CAA51900.1
Ad3	11	F N P V Y P Y	AAP31229.1
Ad7	11	F N P V Y P Y	AEC11879.1
Ad35	11	F N P V Y P Y	AAA75331.1
Ad11	11	F N P V Y P Y	AAA42490.1
Ad2	11	F N P V Y P Y	AAP31232.1
Ad5	11	F N P V Y P Y	AAP31231.1
Ad17	11	F N P V Y P Y	ADY18445.1
Ad37	11	F N P V Y P Y	ABK59080.1
Ad22	11	F N P V Y P Y	BAG69149.1
Ad41(S)	10	F N P V Y P Y	ADN06466.1
Ad41(L)	10	F N P V Y P Y	ADN06467.1

Table S5:

Sequences of penton base protomers used in this study.

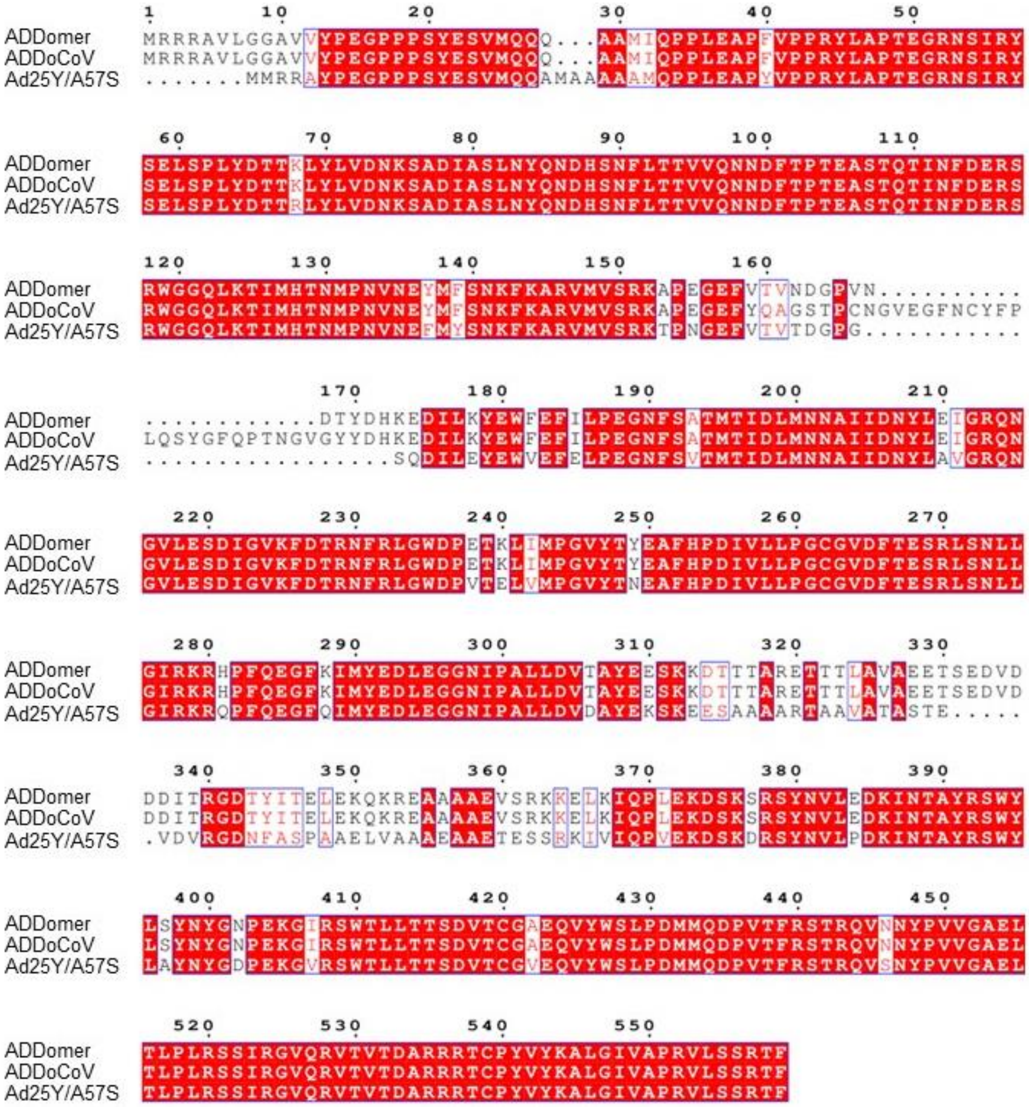

Table S6: Sequences of proteins used for Gigabody preparation.

ID	Primary sequence
Ad25Y ADDomer A57S protomer	MMRRAYPEGPPPSYESVMQQAMAAAAAMQPPLEAPYVPPRYLAPTEGRNSIRYS<br>ELSPLYDTTRLYLVDNKSADIASLNYQNDHSNFLTTVVQNNDFTPTEASTQTIN<br>FDERSRWGGQLKTIMHTNMPNVNEFMYSNKFKARVMVSRKTPNGEFVTVTDGPG<br>SQDILEYEWVEFELPEGNFSVTMTIDLMNNAIIDNYLAVGRQNGVLESDIGVKF<br>DTRNFRLGWDPVTELVMPGVYTNEAFHPDIVLLPGCGVDFTESRLSNLLGIRKR<br>QPFQEGFQIMYEDLEGGNIPALLDVDAYEKSKEESAAAARTAAVATASTEVDVR<br>GDNFASPAAELVAAAEAAETESSRKIVIQPVEKDSKDRSYNVLPDKINTAYRSW<br>YLAYNYGDPEKGVRSWTLLTTSDVTCGVEQVYWSLPDMMQDPVTFRSTRQVSNY<br>PVVGAELLPVYSKSFFNEQAVYSQQLRAFTSLTHVFNRFPENQILVRPPAPTIT<br>TVSENVPALTDHGTLPLRSSIRGVQRVTVTDARRRTCPYVYKALGIVAPRVLSS<br>RTF
ADAH11-Trimer	MKYLLPTAAAGLLLLAAQPAMAQVSKKRARVDDTFNPVYPYDADNAPTVPFINP<br>PFVSSDGFQEKPSGRLVPRGSPGSGYIPEAPRDGQAYVRKDGEWVLLSTFLGGG<br>SQVQLQESGGGLVQAGGSLRLSCAASGDSYTAYVMGWFRQAPGKEREFVASITP<br>TNDNFSYYADSVKGRFTISRDNAKNTVYLQMNSLKPEDTAVYYCALATHSNRPT<br>PITYWGQGTQVTVSSAAASAHHHHHHKLDYKDHDGDYKDHDIDYKDDDDK
Fiber peptide underlaid in orange, T4 foldon in blue, ADAH11 nanobody in green.	

Movies Captions:

Movie S1: Architecture of ADDoCoV candidate vaccine based on cryo-EM data and MD simulations. SARS-CoV-2 RBM derived epitopes (60 copies per ADDoCoV) are colored in red. Pentons forming the nanoparticle scaffold are colored in shades of cyan, lilac and gray.

Movie S2: Gigabody model displaying 12 ADAH11 nanobody trimers**.** Nanobodies (colored in green) are fused to a T4 foldon trimerization domain (blue) and an Adenovirus AD25Y fiber tail peptide (orange). Pentons are colored in shades of cyan, lilac and gray.
